# Vanguard is a Glucose Deprivation‐Responsive Long Non‐Coding RNA Essential for Chromatin Remodeling‐Reliant DNA Repair

**DOI:** 10.1002/advs.202201210

**Published:** 2022-09-01

**Authors:** Ben Zhang, Rick Francis Thorne, Pengfei Zhang, Mian Wu, Lianxin Liu

**Affiliations:** ^1^ Department of Hepatobiliary Surgery The First Affiliated Hospital of USTC Division of Life Sciences and Medicine University of Science and Technology of China Hefei Anhui 230001 China; ^2^ Henan Provincial and Zhengzhou City Key laboratory of Non‐coding RNA and Cancer Metabolism Henan International Join Laboratory of Non‐coding RNA and Metabolism in Cancer People's Hospital of Zhengzhou University Academy of Medical Sciences Zhengzhou University Zhengzhou Henan 450053 China; ^3^ The Cancer Hospital of the University of Chinese Academy of Sciences Institute of Basic Medicine and Cancer (IBMC) Chinese Academy of Sciences Hangzhou Zhejiang 310022 China; ^4^ Anhui Province Key Laboratory of Hepatopancreatobiliary Surgery The First Affiliated Hospital of USTC Hefei Anhui 230001 China; ^5^ Anhui Provincial Clinical Research Center for Hepatobiliary Diseases The First Affiliated Hospital of USTC Hefei Anhui 230001 China

**Keywords:** genomic stability, HMGB1, long non‐coding RNAs, PARP inhibitors, Vanguard

## Abstract

Glucose metabolism contributes to DNA damage response pathways by regulating chromatin remodeling, double‐strand break (DSB) repair, and redox homeostasis, although the underlying mechanisms are not fully established. Here, a previously uncharacterized long non‐coding RNA is revealed that is call Vanguard which acts to promote HMGB1‐dependent DNA repair in association with changes in global chromatin accessibility. Vanguard expression is maintained in cancer cells by SP1‐dependent transcription according to glucose availability and cellular adenosine triphosphate (ATP) levels. Vanguard promotes complex formation between HMGB1 and HDAC1, with the resulting deacetylation of HMGB1 serving to maintain its nuclear localization and DSB repair function. However, Vanguard downregulation under glucose limiting conditions promotes HMGB1 translocation from the nucleus, increasing DNA damage, and compromising cancer cell growth and viability. Moreover, Vanguard silencing increases the effectiveness of poly (ADP‐ribose) polymerase inhibitors against breast cancer cells with wild‐type breast cancer gene‐1 status, suggesting Vanguard as a potential therapeutic target.

## Graphical abstract

A previously uncharacterized long non‐coding RNA Vanguard, responds to glucose deprivation and is essential for DNA repair. Vanguard fundamentally acts to promote complex formation between HMGB1 and HDAC1, with the resulting deacetylation of HMGB1 serving to maintain the nuclear localization and double‐strand break repair function of HMGB1.

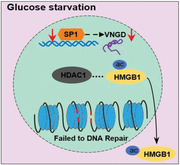

## 1 Introduction

Early studies by Otto Warburg and colleagues revealed the propensity for cancer cells to consume increased quantities of glucose via glycolysis despite the presence of ample oxygen, a phenomenon now called the Warburg effect.^[^ ^1^ ^]^ Compared to oxidative phosphorylation, glycolysis generates adenosine triphosphate (ATP) at a faster rate albeit with lower efficiency, a property that was originally postulated to benefit rapidly proliferating cells.^[^ ^2^ ^]^ However, this reason appears unlikely since numerous studies have indicated mitochondrial generation of ATP remains the primary source for most cancer cells.^[^ ^3^ ^]^ Rather, an alternative view is that high glycolytic rates benefit proliferating cells through the production of glycolytic intermediates, serving as the raw materials for the de novo production of nucleotides, lipids, amino acids, along with contributions to cytosolic Nicotinamide adenine dinucleotide phosphate (NADPH) production via the oxidative pentose phosphate pathway (PPP).^[^ ^4^ ^]^ Indeed, changes in metabolism related to malignancy, broadly known as metabolic reprogramming, have and continue to be extensively researched as vital characteristics of cancer cells.^[^ ^5^, ^6^ ^]^

Another influential 1971 report by Knudsen^[^ ^7^ ^]^ gave rise to the “two‐hit” model, proposing a genetic basis for cancer through recessive mutations in tumor‐initiating genes. Notably, it has been estimated that tens of thousands of damaging events occur daily in the DNA of each human cell,^[^ ^8^ ^]^ invoking the actions of DNA damage response (DDR) pathways to ensure genome integrity. Nevertheless, rather than competing hypotheses between metabolic versus genetic causes of cancer, more contemporary views emphasize the integral links that exist between metabolism and DNA repair.^[^ ^9^ ^]^ Notably, altered metabolism alters redox homeostasis in cancer cells with increases in reactive oxygen species (ROS) causing single strand DNA breaks with replication errors ultimately leading to double stranded DNA breaks (DSBs).^[^ ^10^ ^]^ These lesions are mainly repaired by either error‐free homologous recombination (HR) or error‐prone non‐homologous end‐joining (NHEJ).^[^ ^11^ ^]^ Other destructive metabolites that generate DNA adducts are alkylating agents and aldehydes produced via lipid peroxidation and other metabolic pathways which collectively place an increased burden on the DNA‐repair machinery. Moreover, DNA repair processes are inherently energy‐dependent, relying on ATP supply to drive the unpackaging and remodeling of chromatin to allow DNA repair complexes access to the DSB lesion.^[^ ^12^ ^]^ The available pool of nucleotides also influences DNA repair and thus is also intrinsically linked with metabolism.^[^ ^13^ ^]^ However, the regulatory mechanisms linking cell metabolism with DNA repair are not completely understood. But arguably, this knowledge is vital given that many genotoxic anti‐cancer therapies aim to exploit deficiencies in DNA‐repair in the context of highly proliferative cells.

Of relevance here, the high‐mobility group box 1 (HMGB1) protein belongs to the high‐mobility group gene superfamily which collectively represents the most abundant non‐histone chromatin binding proteins in the nucleus. Intriguingly, HMGB1 fulfills both intracellular and extracellular roles in different pathophysiological contexts.^[^ ^14^ ^]^ Under normal physiological conditions, HMGB1 exists in the nucleus and acts as a chaperone involved in DNA repair processes, telomere maintenance, and chromatin remodeling events along with transcriptional regulation.^[^ ^15^, ^16^, ^17^ ^]^ Alternatively, HMGB1 can also be secreted via active or passive release mechanisms to act as a cytokine, for example, HMGB1 triggers inflammatory responses upon release from activated immune cells or stressed/damaged cells.^[^ ^14^ ^]^ Intracellular HMGB1 has also been linked to cellular energy homeostasis through the promotion of autophagy^[^ ^18^ ^]^ and extracellular HMGB1 can signal through RAGE receptors to promote mitochondrial ATP production.^[^ ^19^ ^]^ Thus, the location of HMGB1 in cells and tissues is critical for determining its function^[^ ^20^ ^]^ and moreover, an extensive list of post‐translational modifications are known to affect its cellular location. For instance, HMGB1 phosphorylation reduces its interaction with the nuclear import protein KAP‐a1, favoring cytoplasmic localization and secretion.^[^ ^21^ ^]^ Different post‐translational modifications are also thought to be involved in eliciting specific HMGB1 functions, for example, the oxidation status of HMGB1 is associated with different secretion mechanisms and functions.^[^ ^22^ ^]^ And while targeting HMGB1 has been suggested for clinical applications, there is the concern that some HMGB1 functions are tumor suppressive in nature such as the direct cytotoxicity toward cancer cells.^[^ ^23^ ^]^ Thus, the key to exploiting HMGB1 may rely on a better understanding of the underlying mechanisms.

Long non‐coding RNAs (lncRNAs) comprise a class of transcripts longer than 200 nucleotides but are not generally translated into proteins. Recent studies indicate that a small but growing list of lncRNAs are crucial involved in maintaining genomic stability.^[^ ^24^, ^25^ ^]^ In this study, we demonstrate that a lncRNA we name Vanguard provides a link between glucose homeostasis, cellular ATP levels, and the maintenance of genome integrity via HMGB1. Manipulating Vanguard levels in cancer cells revealed its essential role in DNA damage repair under both basal and genotoxic stress conditions. We show that Vanguard functions as a scaffold which contains different structural domains supporting the interaction between HMGB1 and histone deacetylase 1 (HDAC1). In turn, this interaction serves to maintain HMGB1 in a deacetylated state to maintain its nuclear localization. Intriguingly, glucose deprivation and associated decreases in cellular ATP results in Vanguard downregulation due to the inhibition of SP1, the transcription factor (TF) primarily responsible for Vanguard transcription. In turn, this undermines the HMGB1–HDAC1 interaction, resulting in the accumulation of acetylated HMGB1 which is transported from nuclear to cytoplasm and lost the function of chromatin remodeling and DNA repair. From the clinical perspective, RNA interference targeting of Vanguard conferred PARP inhibitor (PARPi) sensitivity to human breast cancer cells bearing wild‐type (WT) Breast Cancer gene 1 (BRCA1) expression, suggesting targeting Vanguard has practical utility in synthetic lethal approaches used to target these cancers.

## 2 Results

### 2.1 Identification of LNC8/*AC239868.1* as a Glucose Deprivation‐Responsive lncRNA

**Figure 1:**
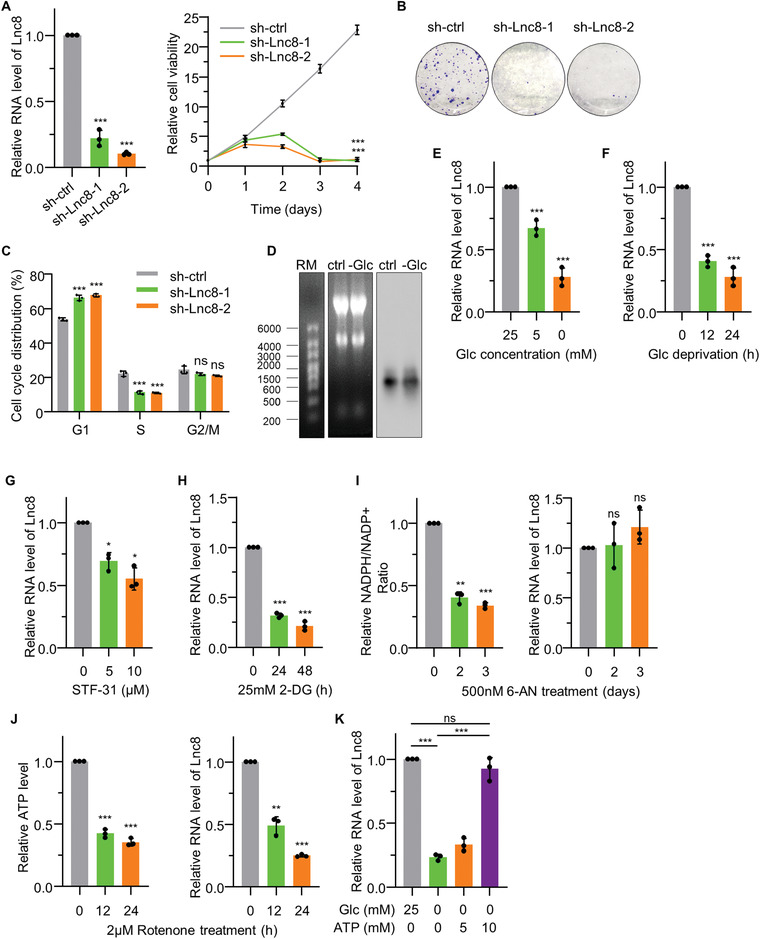
Identification of Lnc8/*AC239868.1* as a glucose deprivation‐responsive LncRNA. A–C) U2OS cells were treated with control shRNA (sh‐ctrl) or independent shRNAs targeting Lnc8 (sh‐8‐1 and sh‐8‐2) before assessing relative cell viability using CCK‐8 assays over 4 days (A), colony formation assays over 2 weeks (B), or cell cycle parameters after 2 days (C). The efficiency of shRNA knockdown of Lnc8 is shown in the left panel of (A). D) Northern blot analysis of Lnc8 in U2OS cells after culture with or without glucose for 24 h. E–H) The relative abundance Lnc8 was determined by qPCR in U2OS cells after (E) culture for 24 h with the indicated glucose concentrations, (F) without glucose for 0–24 h, (G) after 24 h treatment with STF‐31, or (H) after treatment with 2‐DG for 0–48 h. I,J) The relative NADPH/NADP+ ratio in U2OS cells incubated with 500 nm 6‐AN (I) or relative ATP levels after incubation with 2 µm Rotenone (J) for the indicated times. Right panels show relative Lnc8 abundance in the corresponding experiments. K) Relative Lnc8 abundance in U2OS cells after 24 h treatment with the indicated concentration of glucose and ATP. A–K) Results are representative of three independent experiments. Data are mean ± SD, *n* = 3, *p < 0.05; **p < 0.01; ***p < 0.001; ns, not significant, two‐tailed paired Student's *t*‐test.

We initiated this study to identify glucose metabolism‐related lncRNAs or pathways in whose manipulation could be exploited as novel cancer therapeutics. For this, we took advantage of microarray data generated from U2OS osteosarcoma cells subjected to glucose deprivation conditions (Table S1, Supporting Information). From the list of differentially responsive lncRNAs we selected ten previously uncharacterized lncRNAs for further characterization consisting of five upregulated (Lnc1‐5) and five downregulated lncRNAs (Lnc6‐10), respectively. We then validated the expression of each lncRNA in a group of mixed origin cancer cell lines (HepG2 hepatocellular carcinoma, A549 lung cancer and U2OS osteosarcoma; Figure S1A–C, Supporting Information) along with the previously characterized glucose‐responsive gene *STC2*.^[^ ^26^ ^]^ Not all lncRNAs identified by microarray were detected by qPCR but using this approach we arrived at a shortlist of three lncRNAs (Lnc2, Lnc6, and Lnc8) showing differential expression in all three cell lines. Importantly, we found that silencing of Lnc8 produced patent inhibition of cell proliferation in U2OS cells (Figure S1D,E, Supporting Information, and **Figure** **1**A). Moreover, excluding the chance of off‐target effects, depleting Lnc8 expression using two independent shRNAs significantly decreased cellular proliferation as assessed in both CCK‐8 and colony formation assays, which was associated with cell cycle arrest in the G1 phase (Figure 1A–C). We therefore focused our further investigations on Lnc8.

Lnc8 corresponds to the *AC239868.1* annotation present on human chromosome 1 (149861271–149862504). The *AC239868.1* gene is juxtapositioned in a head‐to‐tail orientation with the *H4C15* gene, with the next nearest neighboring gene being *H3C15* lying ≈7 kb upstream of Lnc8 (Figure S1F, Supporting Information). Notably, searches of the mouse genome did not find transcripts with significant homology to *AC239868.1*. Given previous results showing that lncRNAs often influence the expression of their neighboring genes,^[^ ^27^ ^]^ we evaluated the impact of Lnc8 expression on the levels of *H4C15*. However, knockdown of Lnc8 failed to affect *H4C15* mRNA levels (Figure S1G, Supporting Information), while conversely, knockdown of *H4C15* also did not alter Lnc8 levels (Figure S1H, Supporting Information), indicative that no co‐regulatory relationship exists. Northern blotting analysis conducted in U2OS cells detected one major Lnc8 transcript resolving at ≈1.25 kb which was diminished under glucose deprivation conditions (Figure 1D). Notably, the size of this transcript was consistent with the predicted one exon 1234 bp transcript annotated as ENST00000577853.1 in the Ensembl database. Absolute quantitation of Lnc8 levels showed that its basal expression was ≈90, 40, and 160 copies per cell for the U2OS osteosarcoma, PLC/PRF/5 hepatocellular, and MCF‐7 breast cancer cell lines, respectively (Figure S1I, Supporting Information). Last, to ensure our findings were specific, assays employing another two independent primer sets confirmed that Lnc8 levels were responsive to glucose deprivation and could be diminished after targeting with specific shRNAs (Figure S1J,K, Supporting Information).

Following on from this analysis, we sought to further explore the relationship between Lnc8 expression, glucose availability, and overall cellular energetics. As anticipated from the screening data, we observed that Lnc8 expression was downregulated in U2OS cells with reduced glucose levels and with increasing time of glucose deprivation (Figure 1E,F). Consistently, treating U2OS cells with STF‐31, an inhibitor of the major glucose transporter GLUT1^[^ ^28^ ^]^ showed there were dose‐dependent inhibitory effects on Lnc8 levels (Figure 1G). Next to reveal why glucose levels are important for maintaining Lnc8 expression, we considered the principal cellular fates of glucose using an inhibitor‐based strategy. First, we observed that targeting of hexokinase with 2‐deoxy‐d‐glucose (2‐DG) reduced the levels of Lnc8 (Figure 1H), proposing that Lnc8 expression was sensitive to decreased glycolytic flux and/or reductions in the PPP. Instructively, targeting NADPH production which is mainly generated through the PPP^[^ ^29^ ^]^ using 6‐aminonicotinamide, an inhibitor of the NADP+‐dependent enzymes,^[^ ^30^ ^]^ failed to affect Lnc8 levels (Figure 1I). However, treating U2OS cells with rotenone to inhibit ATP synthesis from oxidative phosphorylation,^[^ ^31^ ^]^ resulted in significant decreases in Lnc8 (Figure 1J). Moreover, under glucose deprivation conditions, supplementing culture media with exogenous ATP served to restore the basal levels of Lnc8 (Figure 1K). Thus, collectively these data establish that Lnc8 was responsive to glucose deprivation conditions because of decreases in cellular ATP levels.

On the basis of further investigations disclosed below, we uncovered that the growth inhibitory phenotype resulting from Lnc8/*AC239868.1* knockdown resulted from defective DNA damage repair responses. To provide a more suitable name for common use, we hereafter refer to this gene as the lncRNA Vanguard (abbreviated VNGD).

### 2.2 Vanguard Is Essential for Chromatin Remodeling‐Dependent Genomic DNA Repair

**Figure 2:**
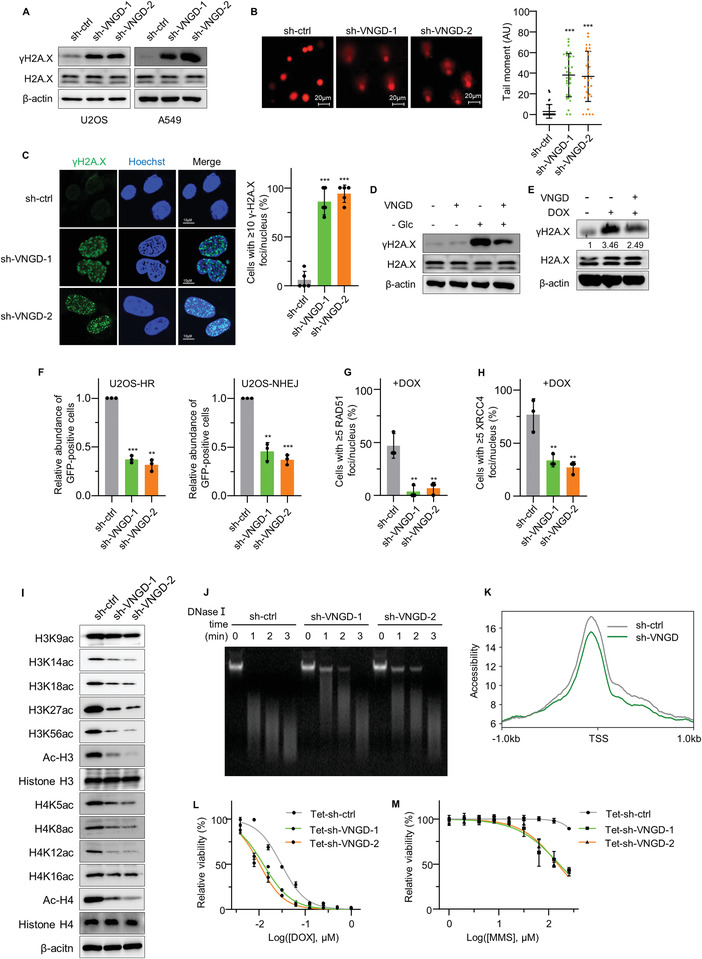
Vanguard is essential for chromatin remodeling‐dependent genomic DNA repair. A) Western blotting to determine the levels of *γ*H2A.X in U2OS (left) and A549 (right) cells following the silencing of VNGD using two independent shRNAs. B) DNA damage accumulation in U2OS cells with or without silencing of VNGD measured using comet assays (left), with tail moments analyzed using CaspLab software (right, *n* = 30, AU: arbitrary units). C) DNA damage accumulation in U2OS cells with or without silencing of VNGD measured using the *γ*H2A.X immunostaining (left), the percentage of cells with ≥10 *γ*H2A.X foci/nucleus was analyzed in random 10 cells (*n* = 5). D,E) Control (pCDH‐ctrl) or VNGD overexpressing (pCDH‐VNGD) U2OS cells were cultured with or without glucose for 24 h (D) or after 0.5 µm DOX treatment for 12 h (E) and the levels of *γ*H2A.X measured by western blot. F) HR/NHEJ reporter assays were performed in U2OS‐HR and U2OS‐NHEJ cells transduced with a control shRNA or with shVNGD‐1 or 2 to silence VNGD expression. Relative abundance of GFP‐positive cells in RFP‐positive U2OS‐HR (left) or U2OS‐NHEJ (right) as determined by flow cytometry. G) U2OS cells with or without VNGD silencing were treated for 4 h with 1 µ DOX before further addition of 10 µm EdU for 2 h and immunostaining against Rad51 and *γ*H2A.X in combination with EdU labeling. Ten random EdU‐positive cells were examined for DNA damage foci positive for Rad51 and *γ*H2A.X with the results reported as the percentage of cells with ≥5 Rad51 foci/nucleus. H) DOX‐treated U2OS cells as per (G) were subjected to immunostaining against XRCC4 and *γ*H2A.X. Ten random cells were examined for DNA damage foci positive for XRCC4 and *γ*H2A.X with the results reported as the percentage of cells with ≥5 XRCC4 foci/nucleus. I) The levels of histone H3 and H4 acetylation measured by western blot in U2OS cells from (A). J,K) Chromatin accessibility measured by the time gradient‐DNase1 hydrolysis assay (J) or ATAC sequencing analysis (K, *n* = 1) in U2OS cells from (A). L,M) U2OS cells were engineered for shRNA‐mediated knockdown of VNGD under the control of a tetracycline responsive promoter (Tet‐sh‐VNGD‐1 and Tet‐sh‐VNGD‐2; Tet‐sh‐ctrl as negative control). The relative cell viability was determined using CCK8 assays after 4 days of treatment with the indicated concentrations of DOX (L) or MMS (M) with 1 µg mL^−1^ DOXY. A–M) Results are representative of three independent experiments except as individually marked. Data are mean ± SD, n = 3, *p < 0.05; **p < 0.01; ***p < 0.001; ns, not significant, two‐tailed paired Student's *t*‐test.

Our preliminary characterization of U2OS cells after VNGD knockdown for 3 days showed obvious increases in micronuclei formation (Figure S2A, Supporting Information) which can indicate defects in the DDR.^[^ ^32^ ^]^ Moreover, further enticing parallels were noted between the ATP‐dependency of VNGD and that of chromatin‐remodeling complexes engaged during DSB repair.^[^ ^33^ ^]^ To evaluate this notional link, we employed western blotting to measure the levels of serine 139‐phosphorylated histone H2AX (*γ*H2AX), a hallmark epigenetic change occurring in the vicinity of DSB.^[^ ^34^ ^]^ Indeed, knockdown of VNGD in U2OS and A549 cells increased the levels of *γ*H2AX (**Figure** **2**A). In line with these findings, comet assays after VNGD knockdown in U2OS cells revealed significant increased lengths of “DNA tails” (Figure 2B), which indicated the increased DNA damage levels. Furthermore, VNGD knockdown resulted in the formation of *γ*H2A.X foci as detected by immunofluorescence staining (Figure 2C). Moreover, overexpression of VNGD attenuated the accumulation of *γ*H2A.X induced by glucose deprivation or Doxorubicin (DOX) treatment (Figure 2D,E, Figure S2B, Supporting Information). We then considered whether VNGD was involved in either or both HR and NHEJ, the two major DSB repair pathways.^[^ ^35^, ^36^ ^]^ Toward this we employed the U2OS‐HR and U2OS‐NHEJ green fluorescent protein (GFP)‐reporter cell lines that provide a measure of the HR and NHEJ repair pathways, respectively.^[^ ^24^ ^]^ Notably, depletion of VNGD in both U2OS‐HR and U2OS‐NHEJ lines resulted in marked reductions in the number of GFP‐positive cells (Figure 2F and Figure S2C, Supporting Information), indicative of the participation of VNGD in both NHEJ and HR. Alternative assays were also conducted to support these findings, measuring XRCC4 that represents one of several core proteins involved in foci formation during NHEJ together with hallmark Rad51 foci that signify S/G2 phase‐specific HR repair.^[^ ^37^, ^38^ ^]^ Consistently, VNGD silencing in U2OS cells reduced DOX‐induced XRCC4 foci along with RAD51 foci in S/G2 phase cells (Figure 2G,H and Figure S2D,E, Supporting Information). Together these results propose that VNGD expression was required for DDRs involving DSB repair under both basal and genotoxic stress conditions.

Changes or exchanges of histones or nucleosome sliding along DNA increases chromatin accessibility and such remodeling of chromatin structures is essential for transcriptional regulation as well as DNA repair processes.^[^ ^39^ ^]^ Various lysine (K) residues in histones H3 and H4 become post‐translationally modified by acetylation with the relative levels revealing the openness of nucleosomes.^[^ ^40^ ^]^ We predicted that VNGD may affect chromatin accessibility and indeed, silencing of VNGD inhibited histone H3 acetylation at K14, K18, K27, and K56, and histone H4 at K5, K8, and K12 to various extents, while the overall levels of H3 and H4 were not affected (Figure 2I). Consistently, DNase I sensitivity assays revealed that chromatin was more resistant to DNase I when VNGD was silenced in U2OS cells (Figure 2J), indicating less nucleosome‐free regions in the absence of VNGD. Independent evidence using ATAC sequencing (ATAC‐seq) and metagene analysis also revealed an overall reduction in chromatin accessibility upon VNGD knockdown (Figure 2K). Notably, consistent with previous research,^[^ ^41^, ^42^ ^]^ depriving U2OS and A549 cells of glucose for 24 h increased the levels of *γ*H2AX, while decreasing the level of acetylated H3 and H4 (Figure S2F, Supporting Information). Since VNGD silencing phenocopied these changes, these data suggest that the downregulation of VNGD upon glucose starvation is a potential causal mechanism underpinning the glucose‐mediated effects on chromatin accessibility and DSB repair processes.

Following on from these findings, we next explored how VNGD was involved in responses to DNA damaging agents. First, we assessed whether such drugs invoked temporal changes in VNGD expression, either at the occurrence of DSBs or otherwise at the initiation of DNA repair. However, treating U2OS cells with DOX and cisplatin (CDDP) that follow fundamentally different damage‐repair time courses showed that the expression of VNGD remained stable (Figure S2G,H, Supporting Information). Thus, VNGD expression appears not to be modulated during the DDR.

We then considered whether VNGD was involved in chromatin remodeling‐dependent or independent DNA repair processes. For this, we treated cells with DOX, methyl methanesulfonate (MMS), and camptothecin (CPT) and measured the effects of these drugs combined with VNGD knockdown on cell proliferation. Notably, the restoration of DSBs induced by DOX relies on HR and NHEJ, which require chromatin remodeling whereas MMS‐related damage necessitates base excision and nick generation, necessitating nucleosome sliding to provide accessibility to damaged bases. Moreover, the repair of CPT‐induced DNA damage was reported to be chromatin remodeling independent due to CPT trapping the TOP1 enzyme on already nicked DNA.^[^ ^43^, ^44^ ^]^ To execute these experiments we employed a Tet (doxycycline [DOXY])‐inducible knockdown system in U2OS cells since direct knockdown of VNGD results in the inhibition of cell proliferation. Important, DOXY treatment itself does not influence VNGD expression or the viability of U2OS cells (Figure S2I, Supporting Information). The results of these assays showed that induction of VNGD increased the sensitivity of U2OS cells to DOX and MMS but not CPT (Figure 2L,M and Figure S2J,K, Supporting Information). Together with the preceding findings, these data show that VNGD is essential for DNA repair under both basal and genotoxic stress conditions with involvement in chromatin remodeling‐reliant DDR pathways.

### 2.3 Vanguard Promotes DNA Repair through Interaction with HMGB1

Many lncRNAs regulate their target genes as transcriptional regulators.^[^ ^45^ ^]^ A logical inference from our preceding findings was that VNGD influenced DSB repair through regulating genes involved in glucose metabolism and/or DDRs. To test this hypothesis, comparative RNA sequencing and gene set enrichment analysis of U2OS cells after VNGD silencing revealed no associations between the differentially expressed genes and defined KEGG gene sets related to glucose metabolism (including glycolysis and gluconeogenesis, the tricarboxylic acid cycle, oxidative phosphorylation, and the PPP [Figure S3A, Supporting Information]). Moreover, there was no significant changes recorded in the mRNA levels of DDR and repair factors compiled by Wood and Lower^[^ ^46^, ^47^ ^]^ (fold change > 2, −*p*‐value < 0.05) in RNA sequencing results (Table S2, Supporting Information).

**Figure 3:**
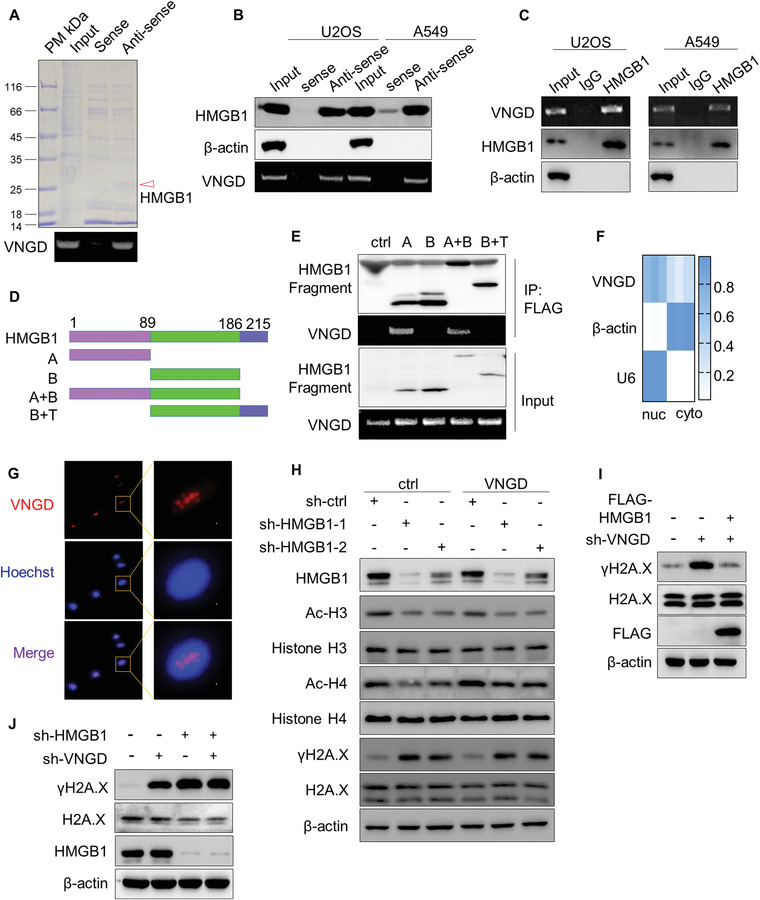
Vanguard promotes DNA repair through interaction with HMGB1. A) RNA–protein pull‐down assays were conducted against U2OS cell lysates with biotin‐labeled sense (control) or antisense (test) VNGD probes. The arrowed band on the Coomassie stained PAGE gel shows a protein identified by mass spectrometry as HMGB1. B,C) RNA pull‐down assays against VNGD (B) or RNA immunoprecipitation (RIP) assays using IgG or HMGB1 antibodies (C) were conducted in U2OS and A549 cells as indicated. The recovery of HMGB1 and VNGD were analyzed by western blotting and RT‐PCR, respectively, with *β*‐actin used as a negative control for western blotting. D,E) Schematic representation of HMGB1 functional domains and the design of domain‐deletion constructs (D). After stable expression of the FLAG‐tagged constructs from (D) in U2OS cells, RIP assays were performed using anti‐FLAG (M2) beads (E). The recovery of the recombinant HMGB1 fragments and VNGD were determined by western blotting and RT‐PCR, respectively. F,G) U2OS cells were separated into nuclear and cytoplasmic fractions and qPCR assays used to measure relative VNGD expression (F). Results are shown as a heatmap with *β*‐actin and U6 RNAs used as markers for the cytoplasmic and nuclear fractions, respectively. Consistently, FISH detected that VNGD was predominately localized to the cell nucleus of U2OS cells (G). H) The levels of *γ*H2A.X, acetylated‐H3, and acetylated‐H4 was measured by western blot after silencing of HMGB1 with two independent shRNAs in control (pCDH‐ctrl) or VNGD overexpressing (pCDH‐VNGD) U2OS cells. I,J) The levels of *γ*H2A.X measured by western blotting in U2OS cells with knockdown of VNGD in combination with empty vector (pCDH) or overexpression of HMGB1 (pDCH‐FLAG‐HMGB1) (I), or in cells with knockdown of VNGD in combination with sh‐ctrl or shHMGB1 (J). A–J) Results are representative of three independent experiments.

We next explored the alternative notion that VNGD acts through mediating RNA–protein interactions using RNA pull‐down assays to interrogate the VNGD‐protein interactome. SDS‐PAGE gel comparisons showed the most prominent band selectively recovered with the antisense VNGD probe from U2OS cells was an ≈25 kDa protein, later identified as HMGB1 using mass spectrometry (**Figure** **3**A). Western blotting analysis of RNA pull‐down samples from both U2OS and A549 cells verified the selective recovery of HMGB1 with VNGD (Figure 3B) while conversely, RNA immunoprecipitation (RIP) assays confirmed that VNGD could be coimmunoprecipitated with HMGB1 (Figure 3C). In combination with this analysis, we also defined the region of HMGB1 responsible for binding to VNGD using a deletion mapping approach. After subdividing HMGB1 into different truncated constructs based on its three major functional domains (Figure 3D), RIP assays established that VNGD only bound to recombinant Flag‐tagged HMGB1 fragments containing the amino terminal A‐box domain while no binding was found with the B‐box or acidic tail domains (Figure 3E). Together these assays establish HMGB1 as a bona fide binding partner of VNGD.

Since HMGB1 is known to enhance DNA repair and chromatin modification after DNA damage,^[^ ^15^ ^]^ we next explored the relevance of the VNGD‐HMGB1 interaction to the DNA repair response. First, as anticipated from a functional viewpoint, analysis of nuclear and cytoplasmic fractions by qPCR showed a strong nuclear pool of VNGD present within U2OS cells (Figure 3F) while FISH assays revealed strong punctate staining in the cell nucleus (Figure 3G). Notably, like the effect of VNGD knockdown, silencing of HMGB1 in U2OS cells also caused DSB repair inhibition and DNA damage accumulation along with reductions in chromatin accessibility and cell viability (Figure 3H and Figure S3B–F, Supporting Information). Nevertheless, the overexpression of VNGD could not attenuate *γ*H2AX accumulation resulting from HMGB1 silencing (Figure 3H) although ectopically expressed HMGB1 eliminated *γ*H2AX accumulation caused by VNGD silencing (Figure 3I). Moreover, silencing of VNGD in combination with HMGB1 did not result in further accumulation of *γ*H2AX above that of HMGB1 knockdown alone (Figure 3J), indicating that VNGD functions in DNA repair in a HMGB1‐dependent manner. Of further interest, while silencing of VNGD resulted in progressively increasing levels of *γ*H2AX over 1–3 days, the levels of HMGB1 decreased but only after 3 days of VNGD silencing (Figure S3G, Supporting Information). On this basis, it can be concluded that VNGD does not principally affect the protein levels of HMGB1 but rather likely affects the function of HMGB1 during DDRs.

### 2.4 Vanguard Cooperates with HDAC1 to Suppress Acetylation of HMGB1

**Figure 4:**
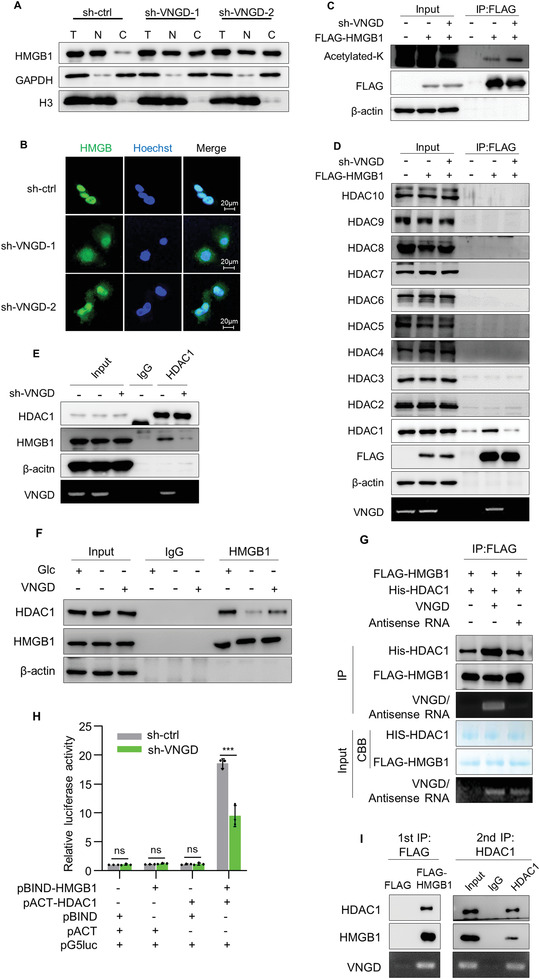
Vanguard cooperates with HDAC1 to suppress acetylation of HMGB1. A) U2OS cells expressing sh‐ctrl, sh‐VNGD‐1, or sh‐VNGD‐2 U2OS cells were separated into nuclear and cytoplasmic fractions and HMGB1 levels measured by western blotting. GAPDH and H3 were used as markers for the cytoplasmic and nuclear fractions, respectively. B) Confocal images of immunostaining against HMGB1 in U2OS cells with or without silencing of VNGD. Hoechst staining was used to decorate cell nuclei. C,D) IP assays were performed using anti‐FLAG (M2) beads against pCDH or pCDH‐FLAG‐HMGB1 expressing U2OS cells in combination with sh‐ctrl or sh‐VNGD expression. Western blotting was used to measure total lysine acetylation (acetylated K) of HMGB1 (C) along with the co‐precipitation of individual HDACs (HDAC1‐10) (D). As indicated, FLAG‐tagged HMGB1 was detected by blotting against FLAG with *β*‐actin serving as a negative control. VNGD was detected in (D) using RT‐PCR. E) RIP assays performed against U2OS cells expressing sh‐ctrl or sh‐VNGD using IgG or anti‐HDAC1 antibodies. Endogenous HMGB1 was detected using western blotting with VNGD detected using RT‐PCR. F) RIP assays performed against control (pCDH‐ctrl) or VNGD overexpressing (pCDH‐VNGD) U2OS cells (with or without glucose for 24 h) using IgG or anti‐HMGB1 antibodies. Endogenous HDAC1 was detected using western blotting and VNGD was detected using RT‐PCR. G) In vitro binding assays were conducted using recombinant sense or antisense VNGD, FLAG‐HMGB1, and HIS‐HDAC1 as indicated. Analyses were performed on FLAG IPs using western blotting against HIS and FLAG, respectively, while inputs for recombinant proteins and VNGD were subjected to Coomassie blue staining and RT‐PCR, respectively. H) Mammalian two‐hybrid assays between pBIND‐HMGB1 and pACT‐HDAC1 in U2OS cells after 48‐h transduction with sh‐ctrl or sh‐VNGD. Results are expressed as relative luciferase activity normalized to the control group using the empty pBIND and pACT vectors. I) Two‐step IP assays conducted in U2OS cells expressing FLAG‐tagged HMGB1. The first‐phase IPs were conducted with FLAG antibodies (left) followed by elution with FLAG peptides. The eluates were further subjected to second‐phase IPs with HDAC1 antibodies (right). Western blotting was performed to detect HMGB1 and HDAC1 using the precipitating antibodies and VNGD was detected using RT‐PCR. A–I) Results are representative of three independent experiments. Data are mean ± SD, *n* = 3, *p < 0.05; **p < 0.01; ***p < 0.001; ns, not significant, two‐tailed paired Student's *t*‐test.

Under normal growth conditions HMGB1 localizes to the nucleus^[^ ^48^ ^]^ and its DNA‐repair‐promoting activity implicitly occurs in the nucleus.^[^ ^15^ ^]^ Consistently we observed that a large proportion of the total cellular HMGB1 protein was found in the nucleus of U2OS cells (**Figure** **4**A,B). However, silencing of VNGD in these cells resulted in a partial redistribution of HMGB1 to a cytoplasmic pool. Intriguingly, depriving cells of glucose also resulted in a partial shift of HMGB1 from the nucleus to the cytoplasm (Figure S4A,B, Supporting Information). Collectively these findings suggested that VNGD may be involved in retaining HMGB1 in the nucleus.

It is known that HMGB1 undergoes post‐translational protein acetylation, and this reversible modification determines its subcellular distribution.^[^ ^48^ ^]^ Lysine acetylation by PCAF, CBP, and p300 serve to direct the localization of HMGB1 to the cytoplasm, while members of the HDAC family act to deacetylate HMGB1 and promote its nuclear retention.^[^ ^48^ ^]^ Thus, we first considered whether VNGD was involved in the regulation of HMGB1 acetylation. Indeed, we found that the total lysine acetylation levels in ectopically expressed Flag‐tagged HMGB1 (FLAG‐HMGB1) increased when VNGD was silenced (Figure 4C). Additionally, prior findings have indicated that the acetylation of two lysine clusters in HMGB1 (K‐28, ‐29, ‐30 and K‐182, ‐183, ‐184, respectively) can regulate HMGB1 subcellular localization.^[^ ^48^ ^]^ On this basis we constructed FLAG‐HMGB1 K to Arginine (R) mutants of each or both clusters (K28‐30R named KR1, K182‐184R named KR2, K28‐30, 182‐184R named KR3). Analysis of the lysine acetylation of each mutant HMGB1 construct after VNGD silencing showed some increased KR1 and KR2 acetylation but no acetylation signals were detected for KR3 (Figure S4C, Supporting Information). These data confirm that the two lysine clusters serve as the major acetylation sites in HMGB1 and that VNGD functions to inhibit the acetylation of both clusters.

We then further explored whether HMGB1 acetylation in the aforementioned sites could inhibit DNA repair. The FLAG‐HMGB1‐KR3 mutant described above was used to mimic HMGB1 in a deacetylated state while each of the lysine residues were substituted with glutamine (Q; FLAG‐HMGB1‐KQ3) to mimic acetylation. Titration of the exogenous HMGB1 constructs showed that the WT and non‐acetylated KR3 FLAG‐HMGB1 could reverse the increased *γ*H2AX levels induced by DOX while FLAG‐HMGB1‐KQ3 had only weak effects (Figure S4D, Supporting Information). Together these data support the notion that acetylation of HMGB1 at these defined sites, serves to inhibit its DNA repair function.

Given the preceding data, we then determined if VNGD cooperated with specific HDACs to maintain HMGB1 in a deacetylated state. We observed that VNGD silencing did not affect the levels of any class I or II HDACs (Figure S4E, Supporting Information), ruling out any involvement of VNGD in maintaining HDAC expression levels. Rather we considered if VNGD affected interactions between specific HDACs and HMGB1. Toward this notion, HDAC1‐10 were individually immunoprecipitated from U2OS cells transduced with Flag‐HMGB1 alone and in combination with knockdown of VNGD. Instructively, only HDAC1 along with VNGD was selectively recovered within Flag‐HMGB1 immunoprecipitates and the amount of recovered HDAC1 was decreased to control levels upon VNGD silencing (Figure 4D). Moreover, the amount of co‐associated HMGB1 within HDAC1 immunoprecipitates was substantially reduced when VNGD was silenced (Figure 4E), suggesting that the role of VNGD is to facilitate the interaction between HDAC1 and HMGB1. Notably, after glucose deprivation for 24 h, the interaction of HMGB1 and HDAC1 was reduced while overexpression of VNGD enhanced their interaction (Figure 4F and Figure S4F, Supporting Information). Supporting this finding, in vitro binding assays and mammalian two‐hybrid assays showed that VNGD increased the association between recombinant HMGB1 and HDAC1 proteins (Figure 4G,H and Figure S4G,H, Supporting Information).

To unequivocally verify that VNGD, HMGB1, and HDAC1 exist within the same complex, we conducted two‐step immunoprecipitation assays in U2OS cells expressing Flag‐HMGB1. As expected, the first‐phase IP with anti‐Flag antibodies recovered HMGB1 along with HDAC1 and VNGD while the second phase IP against HDAC1 showed a similar recovery of HMGB1 and VNGD (Figure 4I), indicating VNGD, HMGB1, and HDAC1 exist as a ternary complex. In consideration of this finding, we examined the predicted structure of VNGD which consists of a bipolar molecule. We then divided VNGD into three fragments, P1 (nt 1–465) and P2 (nt 466–998) forming the “north” and “south” arms along with P3 (nt 999–1234) that aligns toward the middle of the VNGD molecule. Notably, RNA pull‐down assays using the individual VNGD RNA fragments showed that P1 bound to HMGB1 while P2 bound to HDAC1 (Figure S4I, Supporting Information). Together, these findings propose that each pole of VNGD interacts with HMGB1 and HDAC1, respectively, and this complex serves to inhibit HMGB1 acetylation with ensuing effects on its nuclear localization and function.

### 2.5 SP1 Drives Vanguard Transcription under Glucose Replete Conditions

**Figure 5:**
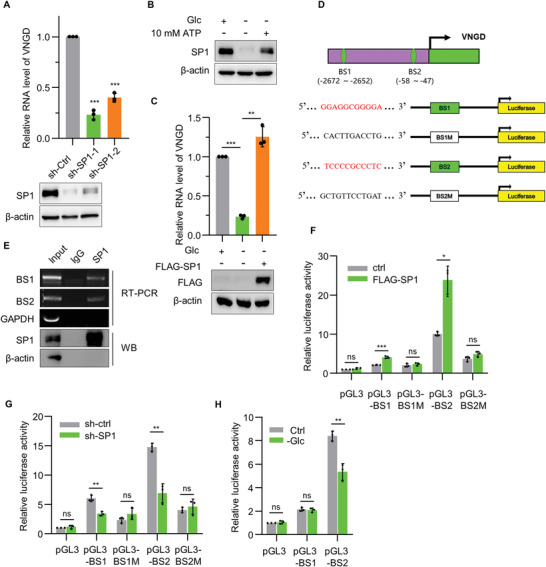
SP1 drives Vanguard transcription under glucose replete conditions. A) The relative expression of VNGD (qPCR, top) and SP1 protein levels (Western blot, bottom) in U2OS cells bearing a control shRNA (sh‐ctrl) or two independent shRNAs targeting SP1 (sh‐SP1‐1 and sh‐SP1‐2). *β*‐actin was used as a loading control throughout. B) Western blot of SP1 levels in U2OS cells cultured with or without glucose for 24 h or without glucose supplemented with exogenous ATP. C) The relative expression of VNGD RNA (qPCR, top) and FLAG‐SP1 protein levels (Western blot, bottom) in U2OS cells transfected with pCDH or pCDH‐FLAG‐SP1 plasmids as indicated and then cultured under glucose deprivation conditions for 24 h. D) Two designated SP1 binding sequences (BS1 and BS2) located in the proximal promoter region of VNGD (top) were used to design pGL3‐based luciferase reporter plasmids containing WT and mutant sequences (bottom). E) ChIP assays conducted against U2OS cells using anti‐SP1 or isotype‐matched antibodies with recovery of SP1 confirmed using western blotting. RT‐PCR was used to amplify bound DNA fragments corresponding to the putative BS1 and BS2 SP1 sites in the VNGD promoter with a GAPDH target sequence employed as a negative control. F–H) The indicated reporter plasmids from (D) were used to transfect U2OS cells in combination with either pCDH‐ctrl or pCDH‐FLAG‐SP1 (SP1) plasmids (F) or lentiviral infection with sh‐ctrl or sh‐SP1 (G). Luciferase reporter assays were then conducted after 12 h or after additional culture with or without glucose for 24 h (H). Values were normalized to the pGL3 control. A–H) Results are representative of three independent experiments. Data are mean ± SD, *n* = 3, *p < 0.05; **p < 0.01; ***p < 0.001; ns, not significant, two‐tailed paired Student's *t*‐test.

We next returned to consider our original observation that glucose deprivation results in decreased levels of VNGD, notionally through transcriptional effects. To shortlist the likely TFs involved, we profiled the proximal promoter region of VNGD using the PROMO database to arrive at 34 predicted TFs which was further refined to 7 high scoring hits from the JASPAR database with predicted promoter binding scores >10 (Figure S5A, Supporting Information). Thereafter, qPCR assays were used to measure the expression changes in each of the 7 TFs in U2OS cells following 24 h of glucose deprivation (Figure S5B, Supporting Information). Among these, only YY1, IFR1, XBP1, and SP1 responded to glucose deprivation but importantly, only SP1 knockdown resulted in decreased levels of VNGD (**Figure** **5**A and Figure S5C–E, Supporting Information). Together these findings prioritized SP1 for further investigation.

Western blotting analysis confirmed that SP1 protein levels were decreased following glucose deprivation but notably SP1 expression could be rescued by the addition of exogenous ATP (Figure 5B). Additionally, the introduction of Flag‐tagged SP1 could similarly restore the expression of VNGD in U2OS cells under glucose deprivation (Figure 5C). As further verification that SP1 acts directly at the VNGD promoter, we performed both chromatin immunoprecipitation (ChIP) and luciferase reporter assays. Based on JASPAR database, we identified two putative SP1‐binding sites (BS1: −2748 to −2738 bp and BS2: −20 to −10 bp) upstream of the VNGD transcriptional start site (Figure 5D). Instructively, DNA fragments containing both BS1 and BS2 were recovered in ChIP assays against SP1 (Figure 5E) and moreover, luciferase reporter assays showed that both BS regions were responsive to SP1 (Figure 5F,G). However, the relative contribution of BS2 appeared greater than BS1 and only the BS2 region was responsive to glucose deprivation‐mediated effects on VNGD transcription (Figure 5H). Collectively, these data establish that SP1 transactivation drives VNGD expression under glucose replete conditions.

### 2.6 Depleting Vanguard Confers Sensitivity to PARP Inhibition in Wild‐Type BRCA1 Cells

Inducing DNA damage and targeting DNA damage repair pathways arguably lie at the core of breast cancer treatment. Beyond traditional radiotherapy or chemotherapy, PARPi increase the requirement for BRCA‐dependent HR activity, producing selective toxicity against homologous BRCA1/2‐deficient cells in part by trapping PARP enzymes on chromatin.^[^ ^49^ ^]^ Nevertheless, a significant proportion of breast cancers develop without BRCA1/2 mutations and such patients currently do not benefit from this therapeutic approach.^[^ ^50^ ^]^ In this regard, we found VNGD expression to be significantly elevated in breast cancer tissues compared to normal breast tissues and this was further increased in more advanced cases (Figure S6A, Supporting Information). On this basis, we next considered if VNGD could be exploited as combinatorial target for synthetic lethal approaches, and in particular, whether targeting VNGD could confer PARPi sensitivity in breast cancer cells expressing WT BRCA1.

**Figure 6:**
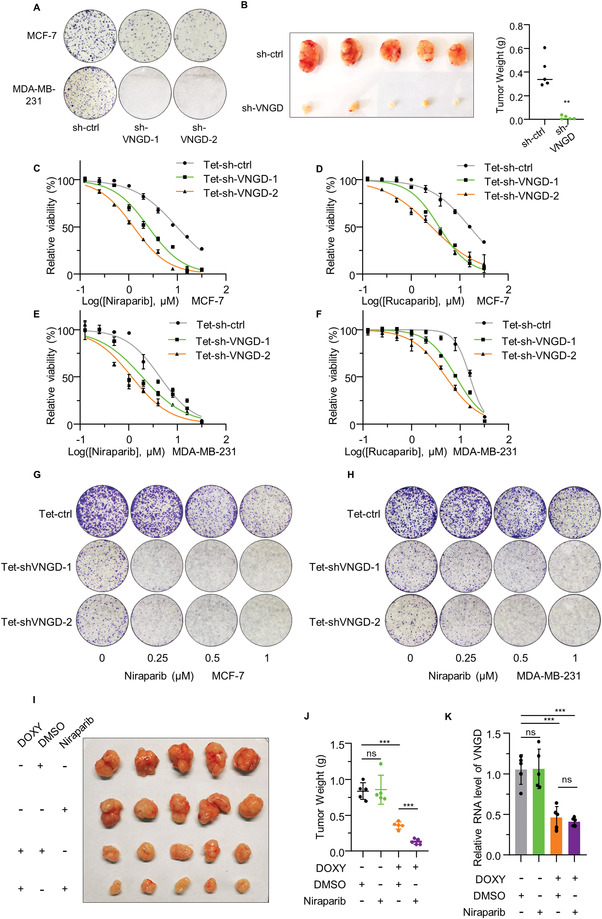
Depleting Vanguard confers sensitivity to PARP inhibition in wild‐type BRCA1 cells. A) Representative colony formation assays conducted in MCF‐7 and MDA‐MB‐231 cells comparing sh‐ctrl transduction against two independent shRNAs targeting VNGD. B) MDA‐MB‐231 cells (5 × 10^6^) transduced with sh‐ctrl or sh‐VNGD were inoculated into the flanks of nu/nu mice (female). After 4 weeks the mice were humanely culled and the xenografted tumors excised, photographed (left), and weighed (right). C,D) The effect of VNGD silencing in combination with Niraparib (C) or Rucaparib (D) were evaluated in sh‐ctrl or two independent sh‐VNGD inducible knockdown MCF‐7 cell lines. Relative cell viability was determined using CCK8 assays after 4 days of treatment with the indicated drug concentrations with 1 µg mL^−1^ DOXY. E,F) The effect of VNGD silencing in combination with Niraparib (E) or Rucaparib (F) were evaluated in sh‐ctrl or two independent sh‐VNGD inducible MDA‐MB‐231 cell lines. Relative cell viability was determined using CCK8 assays after 4 days of treatment with the indicated drug concentrations with 1 µg mL^−1^ DOXY. G,H) Representative colony formation assays conducted in the indicated MCF‐7 (G) or MDA‐MB‐231 (H) cells treated continuously for 12 days with the indicated doses of Niraparib with 1 µg mL^−1^ DOXY. I–K) MDA‐MB‐231 cells (5 × 10^6^) transduced with Tet‐sh‐VNGD were inoculated into the flanks of nu/nu mice (female). After xenograft inoculation for 1 week, a 50 mg kg^−1^ dose of Niraparib or the equal volume of solvent was administered for 2 consecutive days of 3 days using oral gavage, and 2 mg mL^−1^ DOXY was added into drinking water as indicated. After 5 weeks, the mice were humanely culled and the xenografted tumors excised, photographed (I), and weighed (J). The efficiency of DOXY‐induced shRNA knockdown of VNGD in tumors was detected by qPCR (K). A–K) Results are representative of three independent experiments except (B,I–K) (*n* = 5). Data are mean ± SD, *n* = 3, *p < 0.05; **p < 0.01; ***p < 0.001; ns, not significant, two‐tailed paired Student's *t*‐test.

As expected, VNGD silencing resulted in growth inhibition in the MCF‐7 and MDA‐MB‐231 breast cancer cell lines, both bearing WT BRCA1. Colony formation assays showed decreased growth potential of both cell lines, with particularly marked inhibition in the triple‐negative subtype cell line, MDA‐MB‐231 (**Figure** **6**A). This result was also reflected in vivo with tumorigenicity impacted by VNGD silencing when MDA‐MB‐231 cells were grown as xenografted tumors in nu/nu mice (Figure 6B and Figure S6B, Supporting Information). As VNGD knockdown was itself inhibitory, we again turned to the Tet (DOXY)‐inducible knockdown system in order to synchronize the knockdown of VNGD with drug treatments. The veracity of this system was demonstrated where DOXY treatment in the MCF‐7, MDA‐MB‐231, and T‐47D cell lines resulted in substantially depleted VNGD expression (Figure S6C–E, Supporting Information). Analysis of cell proliferation using CCK‐8 assays indicated that VNGD silencing resulted in a strong effect size where the IC50 values of standard chemotherapy drugs (DOX and CDDP) and PARPi (niraparib and rucaparib) were marked decreased in all three cell lines (Figure 6C–F and Figure S6F–M, Supporting Information). Moreover, VNGD silencing also enhanced the ability of PARPi to inhibit colony formation in the MCF‐7, MDA‐MB‐231, and T‐47D cell lines (Figure 6G,H and Figure S6N–Q, Supporting Information). The synergy between targeting VNGD and PARPi was also confirmed in vivo using nu/nu mice where the tumorigenicity of xenografted MDA‐MB‐231 tumors was significantly impacted by induced silencing of VNGD in combination with oral Niraparib (Figure 6I–K and Figure S6R, Supporting Information). Together, these results reveal VNGD as a potential therapeutic target acting in concert with DNA damaging therapeutics including conferring PARPi‐sensitivity to WT BRCA1 breast cancer cells.

## 3 Discussion

Tumor development has been linked to disrupted DDR and repair pathways, while many chemotherapies act by promoting DNA damage, leading to cell death. DNA repair pathways, on the other hand, can help tumor cells survive DNA damage resulting from drug treatment.^[^ ^51^ ^]^ Therefore, combining DDR inhibitors with DNA damaging agents represents a logical strategy.^[^ ^52^, ^53^ ^]^ Toward this notion, combination trials of chemotherapeutics with VX‐970, an inhibitor of the ATR protein responsible for inducing HR DNA repair following replication stress, showed promising responses in chemotherapy‐resistant cancers.^[^ ^52^ ^]^ Tumors also show marked increases in glucose consumption in comparison to non‐proliferating normal tissues^[^ ^1^, ^5^, ^54^ ^]^ and glucose metabolism is involved in DNA repair in a multifarious manner.^[^ ^55^, ^56^, ^57^ ^]^ Targeting glucose metabolism has been highlighted as an alternative therapeutic approach, but with the concern that cancer cells would likely adapt and/or develop resistance to glycolytic inhibitors.^[^ ^58^ ^]^ Therefore, a better understanding of the relationships between glucose metabolism and DDR pathways may help uncover novel vulnerabilities which are open to exploitation. In this regard, we now describe the actions of the lncRNA Vanguard which serves to promote chromatin remodeling‐associated DNA repair.

Glucose deprivation causes acute energy starvation and a shortage of raw materials for biosynthesis, also inducing oxidative stress by decreasing NADPH generation through the PPP^[^ ^59^ ^]^ along with decreasing cellular ATP.^[^ ^60^ ^]^ Amongst various changes there are effects on the DDR,^[^ ^61^ ^]^ with longer term glucose withdrawal resulting in inhibition of DNA repair pathways that can give rise to accumulated levels of DNA damage.^[^ ^42^ ^]^ Consistently we observed that glucose withdrawal increased genome damage in our cell line models with further analysis showing an implicit association with this phenotype and the presence of Vanguard. We also established that Vanguard cellular levels and function were proportional to glucose availability. Notably, not only did Vanguard downregulation or depletion cause DSB accumulation under basal conditions, but we also observed that Vanguard overexpression promoted increased DSB repair following genotoxic stress. Moreover, depressed ATP levels rather than NADPH levels were the primary determining factor of Vanguard levels. Notably, glucose deficiency is known to affect the levels of various TFs and among these, we found that Vanguard expression was selectively dependent on transactivation by SP1. Indeed, prior evidence shows transcriptional inhibition as well as SP1 protein degradation accompanying glucose withdrawal^[^ ^62^, ^63^ ^]^ and consistently, we observed decreases in SP1 mRNA and protein levels under these conditions.

Vanguard was revealed to impact global chromatin accessibility through effects on histone H3 and H4 acetylation and its silencing conferred sensitively to well‐characterized DNA damaging drugs, suggesting a role in chromatin remodeling‐reliant DNA repair processes. Further analysis showed that Vanguard directly binds to HMGB1, known to undertake a plethora of roles in the cell nucleus involving interactions with both DNA and RNA molecules.^[^ ^64^ ^]^ Notably, HMGB1 competes with histone H1 to modify chromatin structure by increasing chromatin mobility.^[^ ^16^ ^]^ Also part of its repertoire, HMGB1 is a putative player in all four major DNA repair pathways, namely nucleotide excision repair, mismatch repair, base excision repair, and DSB repair.^[^ ^65^ ^]^ Functional assays then confirmed that Vanguard promoted DNA DSB repair through the HR and NHEJ pathways. Vanguard is not the only lncRNA reported to participate in DNA repair^[^ ^24^, ^66^ ^]^ but to our best knowledge, is the first reported lncRNA connect cell metabolism with DDR pathways. Similarly, while a number of lncRNAs have been shown to modulate HMGB1 via competing endogenous RNA interactions,^[^ ^67^, ^68^ ^]^ direct interactions occurring with Vanguard are rare. One very recent report showed a brain‐specific lncRNA called BS‐DRL1 interacted with HMGB1 to regulate genome stability in neurons through modulating DNA damage repair.^[^ ^25^ ^]^ The authors did not disclose the underlying mechanisms but argued the case that BS‐DRL1 is involved in cell‐type specific DDRs. In our study, we made a connection between the Vanguard, HMGB1, and the genomic stability of cancer cells.

The levels of HMGB1 are commonly increased in numerous cancer types compared with their parental normal tissues,^[^ ^69^, ^70^, ^71^ ^]^ proposing HMGB1 expression generally acts to promote tumor progression. Considerable emphasis has been paid to the proinflammatory role of secreted HMGB1, while other reports also link HMGB1 cellular distribution with the promotion of tumor cell metabolism. It is therefore intriguing to consider the contextual placement of our findings regarding Vanguard. Under glucose replete conditions, Vanguard orchestrates interactions between HDAC1 and HMGB1 to maintain HMGB1 in a deacetylated state while glucose withdrawal favors HMGB1 acetylation and its relocation to the cytoplasm. Such HDAC‐dependent mechanisms controlling the localization of HMGB1 operate in normal cells including macrophage and hepatocytes,^[^ ^48^, ^72^ ^]^ this appears not to represent an exclusive tumor adaptative mechanism. Indeed, the downregulation of Vanguard and ensuing changes in HMGB1 localization proved a liability with respect to perturbing DNA repair and impacting cancer cell growth and survival. Moreover, exploiting Vanguard to target HMGB1 appears to circumvent concerns of the potential dual roles of HMGB1 in cancer. And we further provide a practical illustration of how Vanguard could be exploited as a targeted therapy in combination with standard chemotherapeutics along with PARPi's.

Regarding the latter, PARPi represent the first clinically approved agents designed to exploit synthetic lethality.^[^ ^73^ ^]^ Multiple clinical trials carried out since 2009 have demonstrated the efficacy of PARPi's against BRCA mutated ovarian and breast cancers with four agents (olaparib, rucaparib, niraparib, and talazoparib) approved by the U.S. Food and Drug Administration and by the European Medicines Agency.^[^ ^74^ ^]^ Moreover, treatment approval for PARPi has now broadened to include recurrent epithelial ovarian, fallopian tube, or primary peritoneal cancers irrespective of BRCA status.^[^ ^75^ ^]^ Nonetheless, there are two primary limitations of these agents, one being that they are unable to significantly impact HR‐proficient BRCA1/2 WT cancer cells and second, similar to numerous chemotherapeutics, PARPi's also face the problem of inherent or acquired drug resistance. Indeed, more than 40% of BRCA mutated ovarian cancer patients failed to benefit from PARPi's.^[^ ^76^ ^]^ Subsequent investigations have identified several resistance mechanisms which in turn have proposed appropriate treatment biomarkers and targets. For example, the chromatin remodeler ALC1 serves to remove inactive PARP1 from chromatin^[^ ^77^ ^]^ and depleting ALC1 re‐sensitized HR‐deficient BRCA1 mutant breast cancer cells to PARPi's.^[^ ^43^ ^]^ Moreover, the combination of ATR and PARP inhibition sensitized resistant ovarian cancer cells by increasing replication fork stalling and DSBs.^[^ ^78^ ^]^ Here we found that knockdown of Vanguard resulted in favorable reductions in the IC50 values of rucaparib and niraparib against various WT‐BRCA1/2 cells (MCF‐7, MDA‐MB‐231, and T‐47D), conferring sensitivity characteristics to PARP inhibition similar to BRCA‐mutant tumors, or so called BRCAness.^[^ ^79^ ^]^ In addition, we were also able to substantiate this concept in vivo where Vanguard knockdown potentiated the activity of Niraparib against human breast cancer xenografts. Collectively, our findings provide proof‐of‐principle data toward further preclinical assessment of Vanguard as therapeutic target. Moreover, with the development of technologies such GapMers^[^ ^80^ ^]^ together with the FDA approval of several siRNA‐based drugs,^[^ ^81^ ^]^ pipelines for achieving the selective targeting of RNAs in the clinical setting are already in place.

## 4 Experimental Section

### Antibodies and Reagents

Table S3, Supporting Information.

### Cell Culture

U2OS, A549, HepG2, MCF‐7, and 293T cell lines were cultured in high glucose (25 mm) Dulbecco's modified Eagle's medium (Gibco) containing 10% fetal bovine serum (Biological Industries) and 1% sodium pyruvate (Gibco) and maintained at 37 °C in a 5% CO2 atmosphere unless otherwise stated. The MDA‐MB‐231 cell line was cultured in Leibovitz's L‐15 (Gibco) without CO_2_.

### RNA Interference and Transfection

Lentiviruses for gene knockdown or overexpression experiments, respectively, were generated by transfecting HEK293T cells with pLKO.1‐based shRNAs, pRev, pGag, and pVSVG (ratio 2:2:2:1) or pCDH‐based complementary DNA (cDNA), pspax2, and pmd2.g (ratio 2:2:1) in Opti‐MEM medium (Gibco) for 48 h (Table S4, Supporting Information). Lentiviral supernatants were 0.45 µm‐filtered, supplemented with polybrene (8 µg mL−^1^) (Sigma Aldrich) before incubating with target cells for 24 h, followed by selection with 5 µg mL^−1^ puromycin. shRNA sequences are shown in Table S5, Supporting Information. Alternatively, transfection of the indicated plasmids (Table S4, Supporting Information) was performed using the Lipofectamine‐3000 reagent (Invitrogen) according to the manufacturer's instructions.

### Quantitative and Semi‐Quantitative RT‐PCR

Total RNA was extracted using TRIzol Reagent (Invitrogen) before synthesis of cDNA using the PrimeScript RT reagent kit (Takara) according to the manufacturer's instructions. Quantitative PCR (qPCR) assays were performed using TB Green real‐time PCR analysis mix (Takara) with the specified primers (Table S6, Supporting Information), and the results were recorded as cycle thresholds (Ct) normalized against the internal control (*β*‐actin). Alternatively, semi‐quantitative RT‐PCR was performed using 2× Taq PCR mix (Vazyme) with 25 cycles for internal controls and 30–40 cycles for lncRNAs.

### Metabolic Measurements

The levels of ATP and NADPH/NADP+ ratios were measured using the ATP (BioVision) and NADPH/NADP+ ratio assays (BioVision), respectively, according to the manufacturer's instructions.

### Absolute RNA Quantitation

Absolute quantitation of RNA expression was performed using the standard curve method. Briefly, full length‐DNA fragments of the target cDNA of interest were used to construct standard curves by plotting Ct values against copy number as determined from the adjusted final concentration of DNA fragments. RNA was extracted from a fixed number of cells and cDNA equivalents from 2000 cells used in each qPCR. Average RNA copy numbers per cell were calculated from the sample Ct values referenced against the linear portion of the standard curve.

### Chemicals Used in this Study

The chemicals used include niraparib (MCE), rucaparib (Selleck), CPT (Selleck), doxorubicin (MCE), cisplatin (MCE), MMS (Sigma), and DOXY (Takara). Cisplatin and DOXY were dissolved in water to yield stock concentrations of 5 mm and 1 mg mL^−1^, respectively. All other compounds were reconstituted in dimethylsulfoxide (DMSO) at 1000× the required concentration, such that the final percentage of DMSO was 0.1%. To all untreated controls, a final concentration of 0.1% DMSO was added.

### Cytosolic/Nuclear Fractionation

Cell suspensions were incubated with hypotonic buffer (25 mm Tris‐HCl pH 7.4, 1 mm MgCl2, 5 mm KCl, 20 nm Leptomycin B) on ice for 10 min before adding an equal volume of hypotonic buffer containing 1% NP‐40 for another 2 min. Homogenates were then centrifuged at 5000 × *g* for 5 min at 4 °C, and the supernatant collected as the cytosol fraction. Pellets were rinsed twice with hypotonic buffer and re‐suspended in nuclear resuspension buffer (20 mm HEPES pH 7.9, 400 mm NaCl, 1 mm EDTA, 1 m EGTA, 1 m DTT, 1 mm PMSF). After incubation on ice for 30 min, the samples were centrifuged at 12 000 × g for 10 min at 4 °C and the supernatant was collected as the nuclear fraction.

### Cell Viability and Colony Formation Assays

Cell viability was measured using the CCK‐8 assay kit (Abcam) according to the manufacturer's instructions. Briefly, cells were seeded at 2 × 10^3^ per well in 96‐well plates overnight before addition of the indicated treatments. After 4 days, 100 µL DMEM containing 10 µL CCK‐8 solution was added and incubated for 30 min under 37 °C before measuring the absorbance at 460 nm. Data were normalized according to the corresponding control (DMSO or ddH_2_O) group. Colony formation assays were conducted over 10–14 days, and cell colonies were stained by crystal violet after fixation with 4% paraformaldehyde.

### Cell Cycle Analysis

Cells were fixed in 70% precooled ethanol at −20 °C for 6 h before washing with PBS and staining with 50 µg mL^−1^ PI containing RNase A (100 µg mL^−1^) for 30 min at room temperature. After PBS washing, cells were subjected to flow cytometry (CytoFLEX, Beckman Coulter) with cell cycle phases determined using CytExpert 2.4 software.

### U2OS‐HR and U2OS‐NHEJ GFP‐Reporter Assays

After lentiviral‐mediated silencing of VNGD or HMGB1 for 24 h, the reporter cell lines U2OS‐HR (carrying integrated DR‐GFP) and U2OS‐NHEJ (carrying integrated EG5‐GFP) were transfected with the I‐SceI‐DsRed plasmid as previously described.^[^ ^82^, ^83^ ^]^ After 36 h, cells were subjected to flow cytometry analysis (CytoFLEX, Beckman Coulter) before calculating the ratio of GFP positive cells in RFP (DsRed) positive cell populations using CytExpert 2.4 software. The relative abundance of GFP‐positive cells was reported from three independent experiments.

### Immunostaining for RAD51 and XRCC4 Foci

After fixation and washing with PBST, cells were incubated with the specified combinations of primary antibodies diluted according to the manufacturer's recommendations before subsequent incubation with the corresponding species‐matched Alexa Fluor 488 (green), 555 (yellow), or 647 (red) conjugated secondary antibodies for 1 h at room temperature. Cell nuclei were then labeled with DAPI as indicated. RAD51 immunostaining was combined with EdU incorporation assays (EdU Cell Proliferation Kit, Beyotime) with signals detected as Alexa Fluor 488 labeling. After mounting, confocal or epifluorescence images were acquired using Zeiss LSM 880 systems, respectively.

### ATAC‐Seq

After harvest and washing with cold PBS, 50 000 cells were gently lysed in 50 µL of cold lysis buffer before centrifugation to yield a nuclear pellet. Thereafter, nuclei were treated with the transposition reaction mix at 37 °C for 30 min (Nextera Tn5 Transposase) before purification and elution into 10 µL elution buffer (10 mm Tris‐HCl, pH 8.0; Qiagen MinElute PCR Purification Kit). Last, adapters were added prior to PCR‐based library amplification. Library quality control was performed using gel electrophoresis and the libraries pooled at equimolar ratios with barcodes and sequenced on the BGISEQ‐500 platform (BGI‐Shenzhen, China). Raw reads were filtered first to remove low‐quality or adaptor sequences by SOAPnuke. Cleaned reads were mapped to the reference genome of GRCh38.p13 using Bowtie2 (version: 2.2.5). MACS2 (version: 2.1.2) was used to call peaks (open chromatin regions) and the chromatin accessibility was analyzed by Deeptools.

### DNase I Chromatin Accessibility Analysis

Chromatin was isolated for 5 min on ice using chromatin buffer (10 mm Tris‐HCl pH 7.5, 5 mm MgCl2, 1 mm CaCl2, 10 mm KCl, 300 mm sucrose, 0.1% Triton X‐100) before washing and resuspension with the same buffer lacking detergent. One‐quarter of the chromatin from a 10 cm plate of cells was digested with DNase I (Thermo Fisher) at three units per 100 µL for the indicated time at 37 °C. Reactions were stopped by adding 10 mm EDTA and incubating for 10 min at 65 °C. DNA was purified using Genomic DNA Purification Kit (Promega) and separated by 1% agarose gel. Visualized images were obtained using Tanon 2500 Imaging Analysis System (Tanon).

### RNA‐Sequencing

1 µg total RNA was captured using Oligo(dT) beads before mRNA fragmentation using divalent cations and high temperature. Priming was performed using random primers and after first and second‐strand cDNA synthesis, the purified double‐stranded cDNAs were repaired at both ends using a dA‐tailing reaction, followed by T‐A ligation to add adaptors to both ends. Size selection of the adaptor‐ligated DNA was then performed using DNA Clean Beads. Each sample was then amplified by PCR using P5 and P7 primers and the PCR products were validated. Thereafter, libraries with different indexes were multiplexed and loaded on an Illumina HiSeq/Illumina Novaseq/MGI2000 instrument for sequencing using a 2 × 150 paired‐end configuration according to manufacturer's instructions. After technical removal of low quality sequences using Cutadapt (V1.9.1), the cleaned data were mapped to the GRCh38.p13 reference genome via Hisat2 (v2.0.1) software. Differential expression analysis was then performed using the DESeq2 Bioconductor package.

### In Vitro Transcription and Biotin RNA Pull‐Down Assay

DNA templates incorporating the T7 RNA polymerase promoter sequence were generated by PCR amplification and then purified using the DNA Gel Extraction Kit (Thermo Fisher). In vitro transcription was performed using the T7‐Flash Biotin‐RNA Transcription Kit (Epicentre) according to the manufacturer's instructions. Biotin RNA pull‐down assays was performed as previously described.^[^ ^27^, ^32^ ^]^ Briefly, 1–2 × 10^7^ cells were lysed in 1.0 mL of RIP buffer (50 mm Tris‐HCl pH 7.5, 150 mm NaCl, 2.5 mm MgCl2, 1 mm EDTA, 5% glycerol, 0.5% NP‐40, 1 mm DTT, supplemented with Protease Inhibitor Cocktail (Roche), 40 U mL^−1^ RNase inhibitor, and 20 µm MG132). Cell lysates were then incubated with streptavidin beads (Invitrogen) coated with the biotin‐labeled RNA or DNA probes (3 µg) at 4 °C for 4 h overnight. Beads were washed five times in RIP buffer (without glycerol) and eluted in Laemmli buffer. The samples were separated by SDS‐PAGE prior to mass spectrometry or western blotting analysis.

### RNA IP and Two‐Step IPs

IP assays were performed as previously described under RNase‐free conditions.^[^^]^ Briefly, 1 × 10^7^–2 × 10^7^ cells were lysed in RIP lysis buffer and incubated with protein A/G beads precoated with the indicated antibodies for 4 h at 4 °C. Thereafter, beads were washed five times using RIP buffer (without glycerol) and the samples were analyzed by western blotting or semi‐qPCR analysis. For two‐step IPs, cell lysates were prepared using lysis buffer (20 mm Hepes pH 7.8, 400 mmm KCl, 5% glycerol, 5 mm EDTA, 5% glycerol, 0.5% NP‐40, 1 mmm DTT, supplemented with Protease Inhibitor Cocktail [Roche], 40 U mL^−1^ RNase inhibitor, and 20 µm MG132). The first IP used anti‐Flag antibodies before elution with 3xFlag peptides. 10% of the sample was reserved for western blotting and semi‐quantitative PCR analysis, while the remaining eluate was subjected to the secondary IP.

### RNA In Situ Hybridization and Immunofluorescence

Cells were fixed on glass coverslips in 4% formaldehyde and permeabilized using 0.1% Triton X‐100 before performing RNA in situ hybridization reactions. In vitro transcribed antisense probes targeting VNGD (Table S7, Supporting Information) were fluorescently labeled using the Nucleic Acid Labeling Kit (Invitrogen) and hybridized with fixed cells as previously described.^[^ ^85^ ^]^ For immunofluorescence, cells permeabilized in 0.2% Triton X‐100 and incubated with primary antibodies overnight at 4 °C and detection performed with Alexa Fluor‐488 secondary antibodies against rabbit IgG.

### Chromatin Immunoprecipitation Assays

ChIP assays were performed according to the manufacturer's instructions (Millipore). Bound DNA fragments were subjected to semi‐quantitative RT‐PCR using the specified primers (Table S6, Supporting Information). Primers amplifying target sequences in glyceraldehyde‐3‐phosphate dehydrogenase (GAPDH) promoters were used as negative controls.

### Tumor Xenograft Model

MDA‐MB‐231 cells expressing control or VNGD shRNA were subcutaneously injected into the dorsal flanks of 4‐week‐old female BALB/c nude mice (Shanghai SLAC Laboratory Animal Co. Ltd.). Mice were humanely euthanized, and tumors were dissected and weighed before analysis. Studies on animals were conducted with approval from the Animal Research Ethics Committee of the University of Science and Technology of China (PXHG‐WM2019062717).

### Northern Blot

10 µg of total RNA was separated on 1% agarose gels before transfer to Hybond‐N membranes (GE) and UV‐cross‐linking. Digoxin‐labeled oligonucleotide probes were generated using the DIG Northern Starter Kit (Roche) according to the manufacturer's instructions. HRP conjugated anti‐digoxin antibody was used to incubate with GE after the hybridization. Visualized images were obtained using Tanon 5200 Imaging Analysis System (Tanon).

### Mammalian Two‐Hybrid Assays

Mammalian two‐hybrid assays were performed as previously reported. Briefly, HMGB1 and HDAC1 cDNA were cloned into the pBIND and pACT vectors, then co‐transfected with the pG5luc vector (Promega). After 48 h, reporter activity was measured using the Luciferase Reporter Assay Kit (Vazyme), and Renilla activity was used to normalize firefly luciferase activity.

### Luciferase Reporter Assays

Luciferase reporter assays were performed according to the manufacturer's instructions (Vazyme). Briefly, U2OS Cells were transfected with the pGL3‐based constructs containing the ABC1 promoter along with Renilla luciferase plasmids under the indicated condition. After 48 h, firefly and Renilla luciferase activity were examined by the Luciferase Reporter Assay System, and Renilla activity was used to normalize firefly activity.

### Comet Assays

Comet assays were performed as described previously using the Comet Assay kit (Trevigen).^[^ ^24^ ^]^

### Recombinant Proteins

Recombinant fla4g‐tagged HMGB1 protein was purified against anti‐flag (M2) beads after expression in 293T cells while His‐tagged HDAC1 was purified against HIS‐Select Agarose Beads after expression in BL21 *Escherichia coli*. The bound proteins were eluted with 3xflag peptides or imidazole, respectively.

### Quantification and Statistical Analyses

No statistical methods were used to predetermine sample size. Statistical analysis was carried out using GraphPad Prism 8 to assess differences between experimental groups. Statistical significance was analyzed by two‐tailed Student's *t*‐test for comparisons of two samples. Tests performed with *p* < 0.05 were considered statistically significant (ns, not significant, *p < 0.05, **p < 0.01, ***p < 0.001)

## Conflict of Interest

The authors declare no conflict of interest.

## Supporting information

Supporting Information

## Data Availability

The data that support the findings of this study are available in the supplementary material of this article.
