# Supplementary material for: Vanguard is a Glucose Deprivation‐Responsive Long Non‐Coding RNA Essential for Chromatin Remodeling‐Reliant DNA Repair
Source: Adv Sci (Weinh). 2022 Sep 1;9(30):2201210. doi: 10.1002/advs.202201210 (PMC9596831; doi:10.1002/advs.202201210)
Supplement: Supplementary file 1 — Supporting Information [file ADVS-9-2201210-s001.pdf]

Supporting Information

Vanguard is a glucose deprivation-responsive long non-coding RNA essential for chromatin remodeling-reliant DNA repair

Ben Zhang, Rick Francis Thorne, Pengfei Zhang, Mian Wu*, Lianxin Liu**

Figure S1

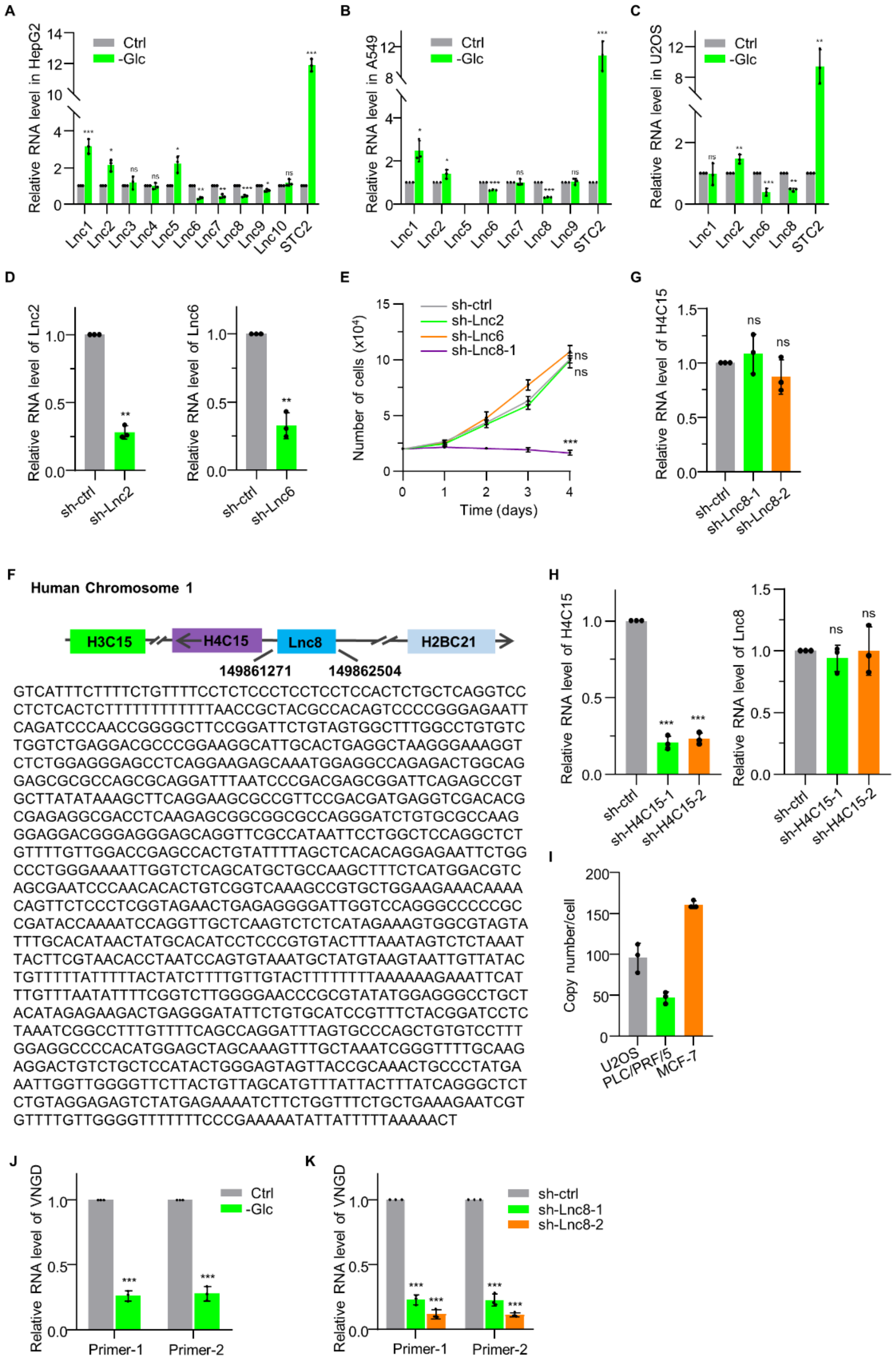

A
Relative RNA level in HepG2
Ctrl
-Glc
Lnc1
Lnc2
Lnc3
Lnc4
Lnc5
Lnc6
Lnc7
Lnc8
Lnc9
Lnc10
STC2
B
Relative RNA level in A549
Ctrl
-Glc
C
Relative RNA level in U2OS
Ctrl
-Glc
D
Relative RNA level of Lnc2
sh-ctrl
sh-Lnc2
Relative RNA level of Lnc6
sh-ctrl
sh-Lnc6
E
Number of cells (x10⁴)
sh-ctrl
sh-Lnc2
sh-Lnc6
sh-Lnc8-1
Time (days)
G
Relative RNA level of H4C15
sh-ctrl
sh-Lnc8-1
sh-Lnc8-2
F
Human Chromosome 1
H3C15
H4C15
Lnc8
H2BC21
149861271
149862504
GTCATTTCTTTTCTGTTTTCCTCTCCCTCCTCCTCCACTCTGCTCAGGTCC
CTCTCACTCTTTTTTTTTTTTTAACCGCTACGCCACAGTCCCCGGGAGAATT
CAGATCCCAACCGGGGCTTCCGGATTCTGTAGTGGCTTTGGCCTGTGTC
TGGTCTGAGGACGCCCGGAAGGCATTGCACTGAGGCTAAGGGAAAGGT
CTCTGGAGGGAGCCTCAGGAAGAGCAAATGGAGGCCAGAGACTGGCAG
GAGCGCGCCAGCGCAGGATTTAATCCCGACGAGCGGATTCAGAGCCGT
GCTTATATAAAGCTTCAGGAAGCGCCGTTCCGACGATGAGGTCGACACG
CGAGAGGCGACCTCAAGAGCGGCGGCGCCAGGGATCTGTGCGCCAAG
GGAGGACGGGAGGGAGCAGGTTCGCCATAATTCCTGGCTCCAGGCTCT
GTTTTGTTGGACCGAGCCACTGTATTTTAGCTCACACAGGAGAATTCTGG
CCCTGGGAAAATTGGTCTCAGCATGCTGCCAAGCTTTCTCATGGACGTC
AGCGAATCCCAACACACTGTCGGTCAAAGCCGTGCTGGAAGAAACAAAA
CAGTTCTCCCTCGGTAGAACTGAGAGGGGATTGGTCCAGGGCCCCCGC
CGATACCAAAATCCAGGTTGCTCAAGTCTCTCATAGAAAGTGGCGTAGTA
TTTGCACATAACTATGCACATCCTCCCGTGTACTTTAAATAGTCTCTAAAT
TACTTCGTAACACCTAATCCAGTGTAAATGCTATGTAAGTAATTGTTATAC
TGTTTTTATTTTTACTATCTTTTGTTGTACTTTTTTTTAAAAAAGAAATTCAT
TTGTTTAATATTTTCGGTCTTGGGGAACCCGCGTATATGGAGGGCCTGCT
ACATAGAGAAGACTGAGGGATATTCTGTGCATCCGTTTCTACGGATCCTC
TAAATCGGCCTTTGTTTTCAGCCAGGATTTAGTGCCCAGCTGTGTCCTTT
GGAGGCCCCACATGGAGCTAGCAAAGTTTGCTAAATCGGGTTTTGCAAG
AGGACTGTCTGCTCCATACTGGGAGTAGTTACCGCAAACTGCCCTATGA
AATTGGTTGGGGTTCTTACTGTTAGCATGTTTATTACTTTATCAGGGCTCT
CTGTAGGAGAGTCTATGAGAAAATCTTCTGGTTTCTGCTGAAAGAATCGT
GTTTTGTTGGGGTTTTTTTCCCGAAAAATATTATTTTTAAAAACT
H
Relative RNA level of H4C15
sh-ctrl
sh-H4C15-1
sh-H4C15-2
Relative RNA level of Lnc8
I
Copy number/cell
U2OS
PLC/PRF/5
MCF-7
J
Relative RNA level of VNGD
Ctrl
-Glc
Primer-1
Primer-2
K
Relative RNA level of VNGD
sh-ctrl
sh-Lnc8-1
sh-Lnc8-2
Primer-1
Primer-2

Figure S1. Screening of glucose-responsive lncRNAs and characterization of Lnc8/AC239868.1

(A-C) qPCR-based validation of glucose-responsive LncRNAs identified by microarray analysis. The relative expression of LncRNAs 1-10 (Lnc1-Lnc10) was determined in (A)HepG2, (B) A549, or (C) U2OS cells after cultured with or without glucose for 24 hours. The absence of any LncRNA on each graph indicates no detectable expression. STC2 mRNA levels were included as a positive control throughout.

(D-E) Silencing of Lnc8 but not Lnc2 or Lnc6 inhibits cell proliferation. U2OS cells were treated with control shRNA (sh-ctrl) or shRNAs targeting Lnc2 or Lnc6 (D) or Lnc8 (refer Figure 1I; sh-8-1) before assessing cell numbers by counting over 4 days (E).

(F) Genomic location of the Lnc8 gene proximal to the H4C15 coding gene (top). Lnc8 transcript sequence (bottom).

(G) U2OS cells were transduced with two independent shRNAs targeting Lnc8 with the relative levels of H4C15 mRNA measured by qPCR.

(H) U2OS cells were transduced with two independent shRNAs targeting H4C15 with the relative levels of H4C15 (left) and lnc8 (right) were measured by qPCR.

(I) Absolute copy number of lnc8 in U2OS, PLC/PRF/5, and MCF7 cells determined using a standard curve-based qPCR method.

(J-K) The relative abundance of Lnc8 was determined by qPCR with 2 independent pairs of primers in U2OS cells after culture with or without glucose for 24h for 24 h (J) or after silencing of Lnc8 with the indicated shRNAs (K).

(A–K) Results are representative of three independent experiments. Data are mean ± SD, $n = 3$, *$p < 0.05$; **$p < 0.01$; ***$p < 0.001$; ns, not significant, two-tailed paired Student's t test.

Figure S2

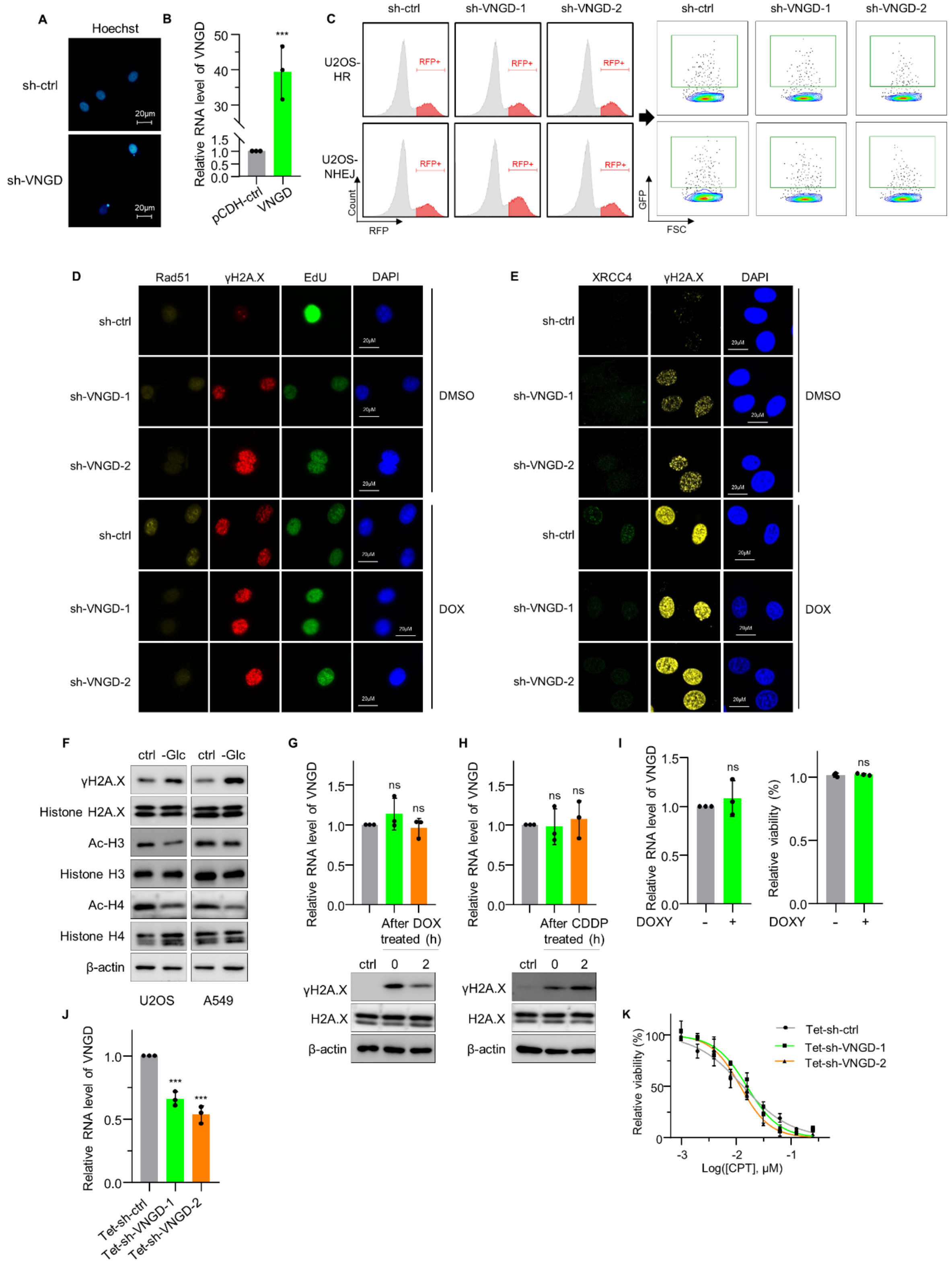

A
Hoechst
sh-ctrl
sh-VNGD
20μm
B
Relative RNA level of VNGD
pCDH-ctrl
VNGD
***
C
sh-ctrl
sh-VNGD-1
sh-VNGD-2
U2OS-HR
U2OS-NHEJ
RFP+
Count
RFP
GFP
FSC
D
Rad51
γH2A.X
EdU
DAPI
DMSO
DOX
E
XRCC4
F
ctrl -Glc
γH2A.X
Histone H2A.X
Ac-H3
Histone H3
Ac-H4
Histone H4
β-actin
U2OS
A549
G
After DOX treated (h)
ns
ctrl 0 2
H2A.X
H
After CDDP treated (h)
I
DOXY
Relative viability (%)
J
Tet-sh-ctrl
Tet-sh-VNGD-1
Tet-sh-VNGD-2
K
Log([CPT], μM)

Figure S2. Supplementary figures related to Figure 2.

(A) Examination of U2OS cell nuclei in control or VNGD knockdown cells using Hoechst staining reveals evidence of increased micronuclei after VNGD silencing for 3 days.

(B) The relative levels of VNGD in control (pCDH-ctrl) or VNGD overexpressing (pCDH-VNGD) U2OS cells as determined by qPCR.

(C) HR/NHEJ reporter assays were performed in U2OS-HR and U2OS-NHEJ cells after transduction with a control shRNA or with shVNGD-1 or 2 to silence VNGD expression. After 24h, the cells were then transfected with the I-SceI-DsRed plasmid for a further 36h before flow cytometric analysis. DsRed-positive (transfected) cells were gated in the RFP channel (left) and this population used to calculate the ratio of GFP-positive cells (right).

(D) Confocal images showing representative U2OS cells with or without silencing of VNGD before and after treating cells for 4h with 1μM DOX to induce DNA damage. The cells were then treated for 2h with 10μM EdU before conducting EdU labeling (green) in combination with immunostaining against Rad51 (yellow) and γH2A.X (red). Counterstaining with DAPI (blue) was used to decorate cell nuclei with positive EdU staining used to identify S/G2 phase cells.

(E) U2OS cells were treated with DOX as per (D) before conducting immunostaining against XRCC4 (green) and γH2A.X (yellow) with counterstaining using DAPI (blue).

(F) Western blotting to determine the levels of γH2A.X, acetylated H3 and acetylated H4 in U2OS (left) and A549 (right) cells after cultured with or without glucose for 24h.

(G-H) U2OS cells were treated for 6h with either 1μM DOX (G) or 10μM CDDP (H). The relative abundance of VNGD was measured using qPCR (top) comparing untreated (ctrl), drug treated (0) and 2h post-treatment recovery (2) cells. The relative levels of unrepaired DNA damage was measured by Western blotting against γH2A.X.

(I) The abundance of VNGD was measured by qPCR after treating the Tet-sh-ctrl U2OS cell line with 1μg ml^{-1} DOXY for 4 days (left) and the relative cell viability was measured using CCK-8 assays (right).

(J) The efficiency of Tet-shRNA knockdown of VNGD was measured by qPCR after being treated with 1μg ml^{-1} DOXY for 4 days.

(K) U2OS cells were engineered for shRNA-mediated knockdown of VNGD under the control of a tetracycline responsive promoter (Tet-sh-VNGD-1 and Tet-sh-VNGD-2; Tet-sh-ctrl as negative control). The relative cell viability was determined using CCK-8 assays after 4 days of treatment with the indicated concentrations of CPT and 1μg ml-1 DOXY.

(A–K) Results are representative of three independent experiments. Data are mean ± SD, n = 3, *p < 0.05; **p < 0.01; ***p < 0.001; ns, not significant, two-tailed paired Student's t test.

Figure S3

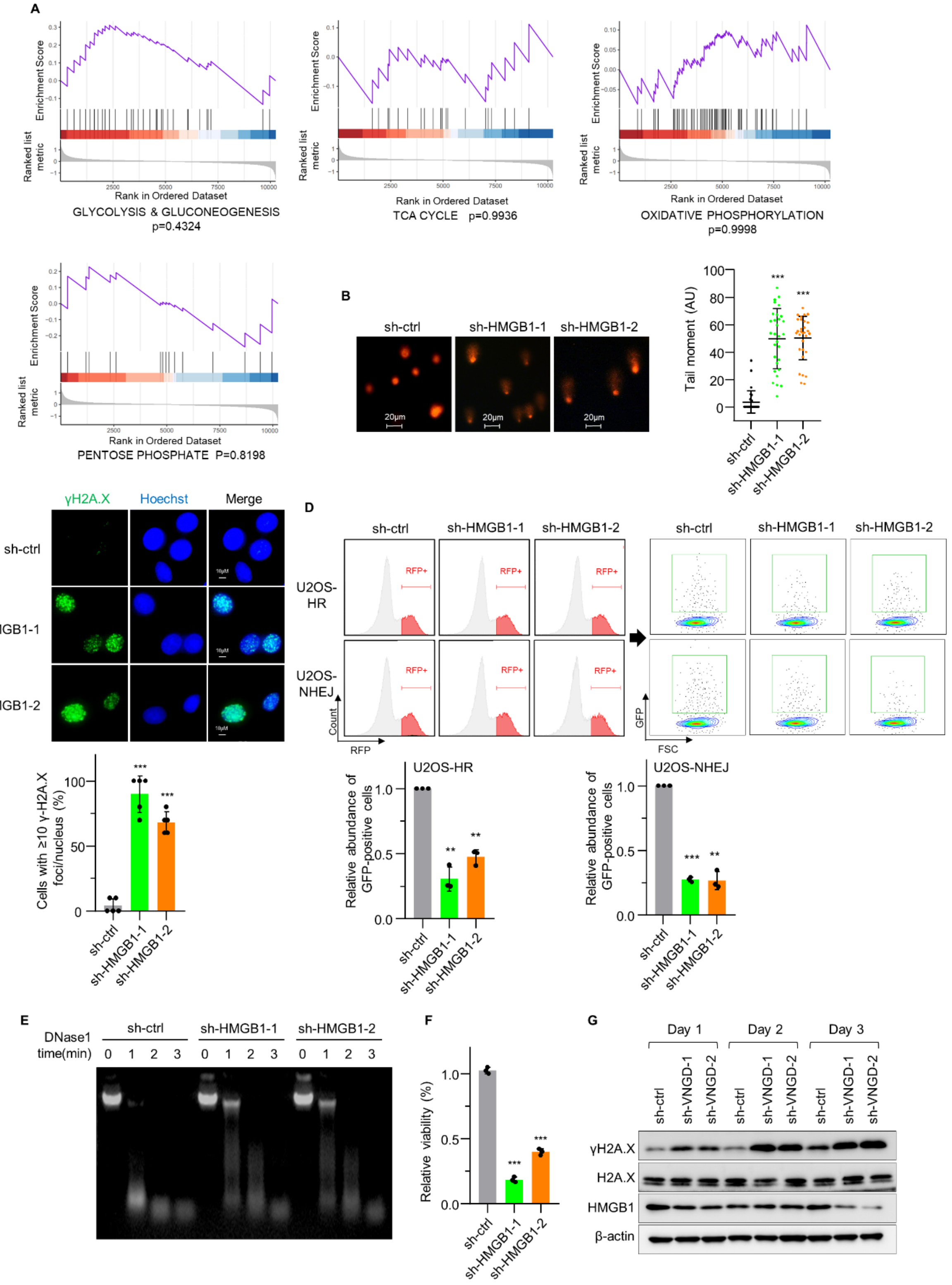
A
Enrichment Score
Ranked list metric
Rank in Ordered Dataset
GLYCOLYSIS & GLUCONEOGENESIS
p=0.4324
TCA CYCLE p=0.9936
OXIDATIVE PHOSPHORYLATION
p=0.9998
PENTOSE PHOSPHATE P=0.8198
B
sh-ctrl
sh-HMGB1-1
sh-HMGB1-2
20μm
Tail moment (AU)
C
γH2A.X
Hoechst
Merge
Cells with ≥10 γ-H2A.X foci/nucleus (%)
D
U2OS-HR
U2OS-NHEJ
RFP+
Count
RFP
GFP
FSC
Relative abundance of GFP-positive cells
E
DNase1 time(min)
F
Relative viability (%)
G
Day 1
Day 2
Day 3
sh-VNGD-1
sh-VNGD-2
γH2A.X
H2A.X
HMGB1
β-actin

Figure S3. Supplementary figures related to Figure 3.

(A) RNA sequencing was performed on U2OS cells following the silencing of VNGD. The differentially expressed genes were compared to the indicated gene sets in KEGG by GSEA.

(B) DNA damage accumulation in U2OS cells with or without silencing of HMGB1 measured using comet assays (left) with tail moments analysed using CaspLab software (right, n=30, AU: arbitrary units).

(C) DNA damage accumulation in U2OS cells with or without silencing of HMGB1 was measured using γH2A.X immunostaining (left) with the percentage of cells with ≥10 γ-H2A.X foci/nucleus was determined in 10 random cells (n=5).

(D) HR/NHEJ reporter assays were performed in U2OS-HR and U2OS-NHEJ cells transduced with the indicated control or HMGB1 targeting shRNAs. After 24h, the cells were then transfected with the I-SceI-DsRed plasmid for a further 36h before flow cytometric analysis. DsRed-positive (transfected) cells were gated in the RFP channel (top left) and this population used to calculate the ratio of GFP-positive cells (top right). The relative abundance of GFP-positive cells in the U2OS-HR and U2OS-NHEJ cells are shown in the bottom left and right, respectively.

(E) Chromatin accessibility measured by the time gradient-DNase1 hydrolysis assay in U2OS cells from (B).

(F) After silencing HMGB1 in U2OS cells with the indicated shRNAs, the cells were cultured another 2 days before assessing relative cell viability using CCK-8 assays.

(G) U2OS cells were transduced with sh-ctrl, sh-VNGD-1 or shVNGD-2 lentiviruses and selected with puromycin for a further 24h. Thereafter, the levels of γH2A.X and HMGB1were measured by Western blotting over three days of culture.

(A–G) Results are representative of three independent experiments except as individually marked. Data are mean ± SD, n = 3, *p < 0.05; **p < 0.01; ***p < 0.001; ns, not significant, two-tailed paired Student’s t test.

Figure S4

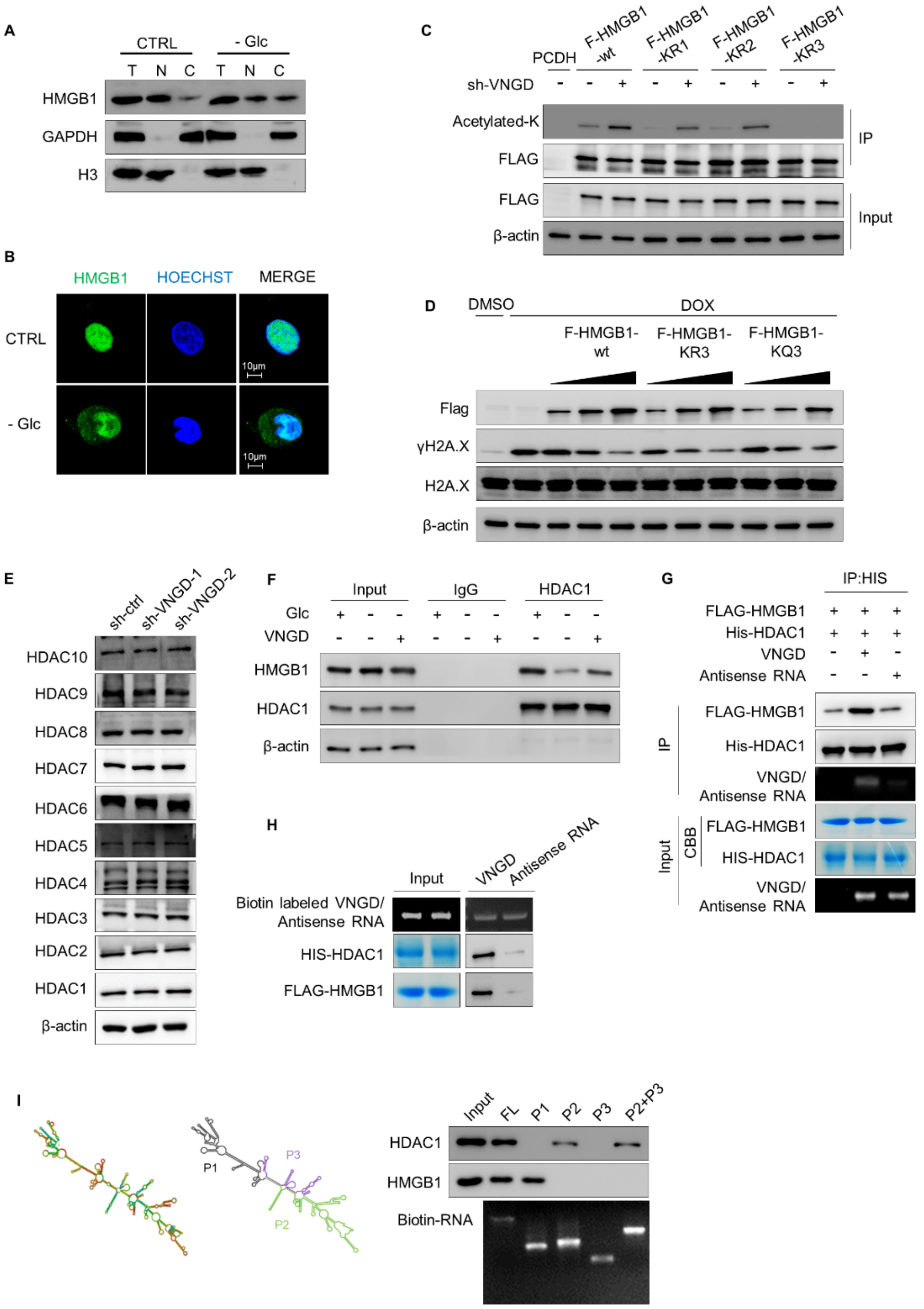
A
CTRL
- Glc
T N C T N C
HMGB1
GAPDH
H3
B
HMGB1
HOECHST
MERGE
CTRL
- Glc
10μm
C
PCDH
F-HMGB1 -wt
F-HMGB1 -KR1
F-HMGB1 -KR2
F-HMGB1 -KR3
sh-VNGD
Acetylated-K
FLAG
FLAG
β-actin
IP
Input
D
DMSO
DOX
F-HMGB1-wt
F-HMGB1-KR3
F-HMGB1-KQ3
Flag
γH2A.X
H2A.X
β-actin
E
sh-ctrl
sh-VNGD-1
sh-VNGD-2
HDAC10
HDAC9
HDAC8
HDAC7
HDAC6
HDAC5
HDAC4
HDAC3
HDAC2
HDAC1
β-actin
F
Input
IgG
HDAC1
Glc
VNGD
HMGB1
HDAC1
β-actin
G
IP:HIS
FLAG-HMGB1
His-HDAC1
VNGD
Antisense RNA
IP
FLAG-HMGB1
His-HDAC1
VNGD/ Antisense RNA
Input
CBB
FLAG-HMGB1
HIS-HDAC1
VNGD/ Antisense RNA
H
Input
VNGD
Antisense RNA
Biotin labeled VNGD/ Antisense RNA
HIS-HDAC1
FLAG-HMGB1
I
P1
P2
P3
Input
FL
P1
P2
P3
P2+P3
HDAC1
HMGB1
Biotin-RNA

Figure S4. Supplementary figures related to Figure 4.

(A) U2OS cells cultured with or without glucose for 24h before separation into nuclear and cytoplasmic fractions. HMGB1 levels measured by Western blotting in total, nuclear and cytoplasmic fractions with GAPDH and H3 used as markers for the cytoplasmic and nuclear fractions, respectively.

(B). HMGB1 immunostaining in U2OS cells cultured with or without glucose as per (A).

(C) IP assays were performed using anti-FLAG (M2) beads against the indicated recombinant proteins expressing U2OS cells in combination with sh-ctrl or sh-VNGD expression. Western blotting was used to measure total lysine acetylation (acetylated K) of the indicated protein.

(D) 293T cells were treated with 1μM DOX for 6h after transfecting 0.5, 1 or 2 μg (left to right) of the indicated plasmids (all lines transfected 2μg plasmids supplemented by PCDH). After 2h post-treatment recovery, the levels of γH2A.X were determined by Western blotting.

(E) Western blotting analysis of HDAC1-10 in U2OS cells bearing control shRNA or VNGD silencing shRNAs. β-actin was used as a loading control.

(F) RIP assays performed against Control (pCDH-ctrl) or VNGD overexpressing (pCDH-VNGD) U2OS cells (with or without glucose for 24h) using IgG or anti-HDAC1 antibodies. Endogenous HMGB1 was detected using Western blotting with VNGD detected using RT-PCR.

(G) In vitro binding assays were conducted using recombinant sense or antisense VNGD, FLAG-HMGB1 and HIS-HDAC1 as indicated. Analyses were performed on HIS IPs using Western blotting against FLAG and HIS, respectively while inputs for recombinant proteins and VNGD were subjected to Coomassie blue staining and RT-PCR, respectively.

(H) Biotin-RNA pull-down assays were conducted using recombinant biotin-labeled sense or antisense VNGD, FLAG-HMGB1 and HIS-HDAC1 as indicated. Analyses were performed using Western blotting against FLAG and HIS, respectively while inputs for recombinant proteins and VNGD were subjected to Coomassie blue staining and RT-PCR, respectively.

(I) Predicted free energy secondary structure of VNGD (left) showing the design of the three fragments designated P1-P3 used for binding analyses (middle). RNA-protein pull-down assays were conducted against U2OS cell lysates using biotinylated recombinant VNGD fragments as indicated (right). Endogenous HDAC1 and HMGB1were detected in the pulldown samples by Western blotting with agarose gel electrophoresis used to show the synthesized products of the different recombinant VNGD fragments.

(A–I) Results are representative of three independent experiments.

Figure S5

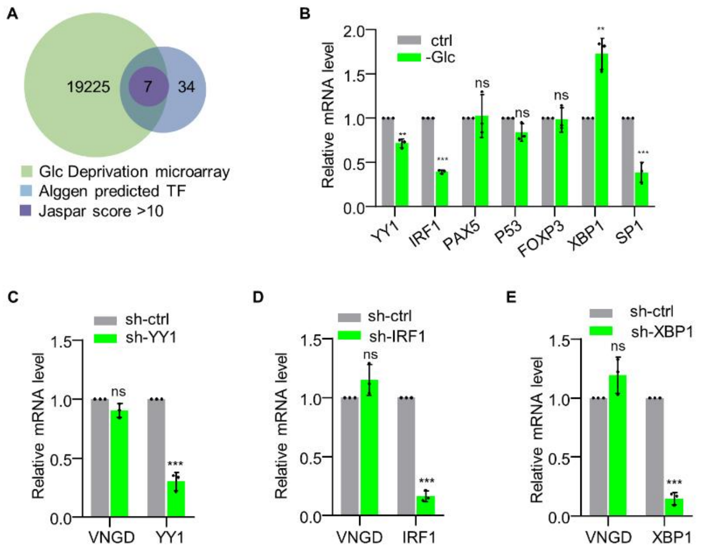

A
19225
7
34
Glc Deprivation microarray
Alggen predicted TF
Jaspar score >10
B
ctrl
-Glc
Relative mRNA level
2.0
1.5
1.0
0.5
0.0
**
***
ns
ns
ns
**
***
YY1
IRF1
PAX5
P53
FOXP3
XBP1
SP1
C
sh-ctrl
sh-YY1
Relative mRNA level
1.5
1.0
0.5
0.0
ns
***
VNGD
YY1
D
sh-ctrl
sh-IRF1
Relative mRNA level
1.5
1.0
0.5
0.0
ns
***
VNGD
IRF1
E
sh-ctrl
sh-XBP1
Relative mRNA level
1.5
1.0
0.5
0.0
ns
***
VNGD
XBP1

Figure S5. Supplementary figures related to Figure 5.

(A) Venn diagram comparing all glucose responsive genes against transcription factors predicted to interact with the VNGD promoter. Data from the PROMO database (34) and high scoring hits from the JASPAR database (promoter binding scores >10; 7) are shown.

(B) Relative mRNA abundance of the 7 indicated transcription factors in U2OS cells after culture with or without glucose for 24 hours determined by qPCR.

(C-E) Assessment of VNGD levels using qPCR (left panels) after shRNA-mediated knockdown of YY1 (C), IRF1 (D), or XBP1 (E) in U2OS cells. Knockdown efficiency was verified by qPCR (right panels).

(A–E) Results are representative of three independent experiments. Data are mean ± SD, n = 3, *$p < 0.05$; **$p < 0.01$; ***$p < 0.001$; ns, not significant, two-tailed paired Student's t test.

Figure S6

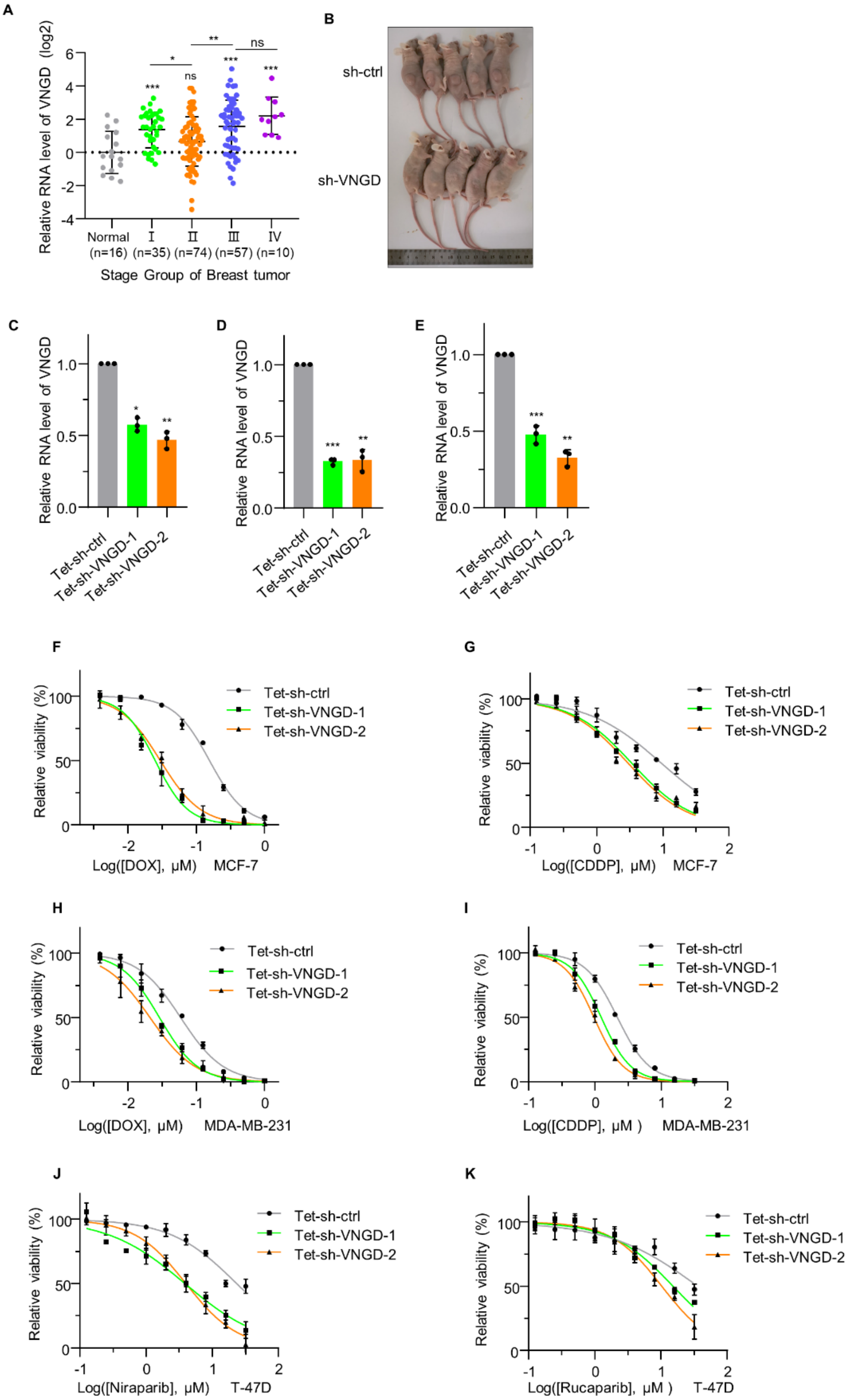
A
B
sh-ctrl
sh-VNGD
Relative RNA level of VNGD (log2)
Stage Group of Breast tumor
Normal (n=16)
Ⅰ (n=35)
Ⅱ (n=74)
Ⅲ (n=57)
Ⅳ (n=10)
C
D
E
Relative RNA level of VNGD
Tet-sh-ctrl
Tet-sh-VNGD-1
Tet-sh-VNGD-2
F
G
H
I
J
K
Relative viability (%)
Log([DOX], μM) MCF-7
Log([CDDP], μM) MCF-7
Log([DOX], μM) MDA-MB-231
Log([CDDP], μM) MDA-MB-231
Log([Niraparib], μM) T-47D
Log([Rucaparib], μM) T-47D

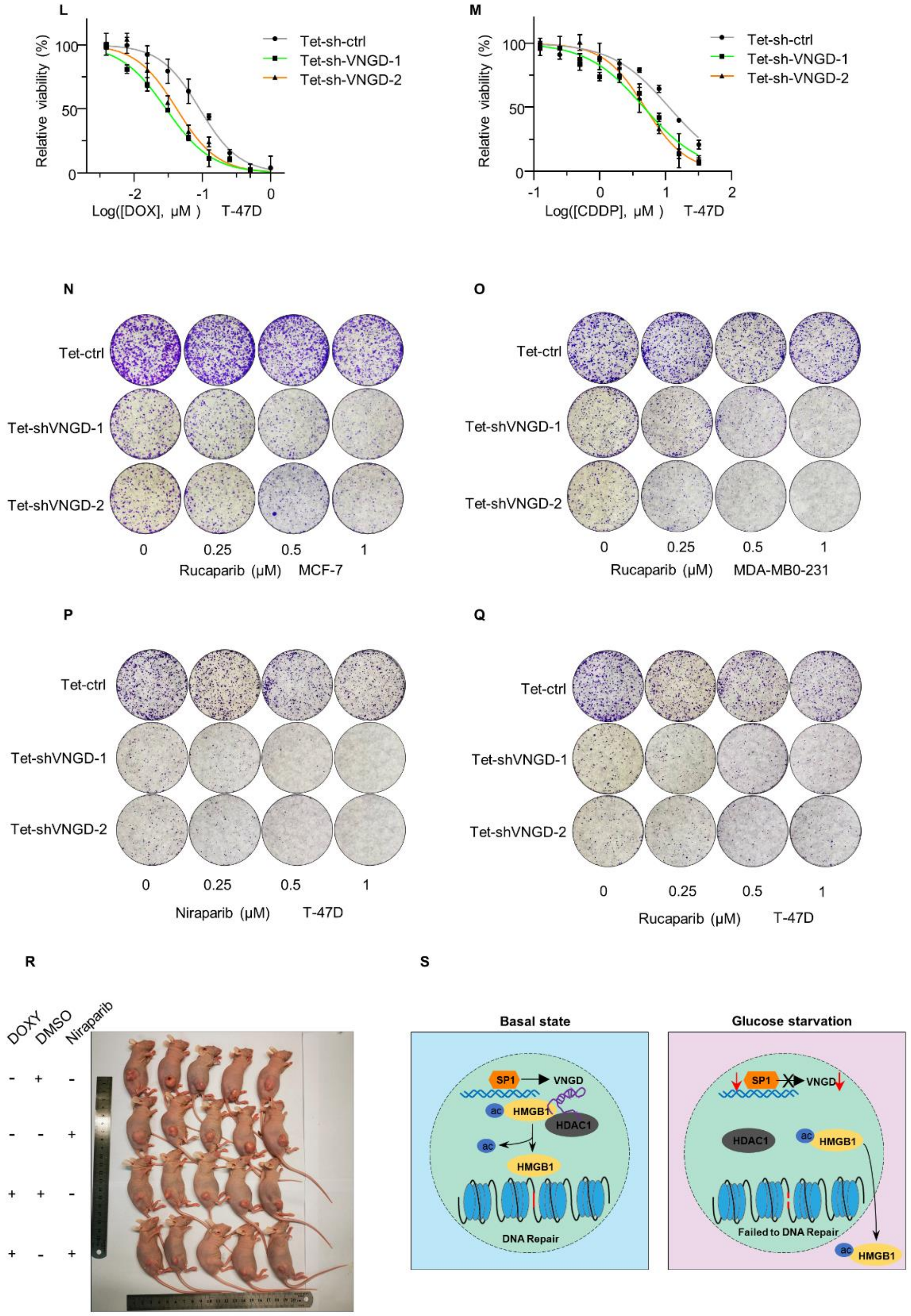

L
Relative viability (%)
100
50
0
-2
-1
0
Log([DOX], μM)
T-47D
Tet-sh-ctrl
Tet-sh-VNGD-1
Tet-sh-VNGD-2
M
Relative viability (%)
Log([CDDP], μM)
T-47D
N
Tet-ctrl
Tet-shVNGD-1
Tet-shVNGD-2
0
0.25
0.5
1
Rucaparib (μM)
MCF-7
O
Rucaparib (μM)
MDA-MB0-231
P
Niraparib (μM)
T-47D
Q
Rucaparib (μM)
T-47D
R
DOXY
DMSO
Niraparib
S
Basal state
SP1
VNGD
ac
HMGB1
HDAC1
DNA Repair
Glucose starvation
Failed to DNA Repair

Figure S6. Supplementary figures related to Figure6.

(A) The RNA levels of VNGD in breast cancer cDNA arrays (OriGene; BCRT101, BCRT102, BCRT103 and BCRT104) were detected by qPCR. Levels were normalized to β-actin and statistical differences determined using the Mann–Whitney test.
(B) Whole body images of mice from Figure 6B shown 4 weeks after xenograft inoculation (n=5).
(C-E) The efficiency of Tet-shRNA knockdown of VNGD in MCF-7 (B), MDA-MB-231 (C) or T-47D (D) was measured by qPCR after 1μg ml-1 DOXY treatment for 4 days.
(F-M) Functional evaluation of VNGD silencing in Tet-inducible knockdown cell lines in combination with clinically relevant agents. Relative cell viability was determined using CCK8 assays after 4 days of treatment with 1μg ml-1 DOXY and the respective doses of the indicated drugs.
(N-Q) Representative colony formation assays conducted in Tet-inducible knockdown MCF-7 and MDA-MB-231 cells (after 12 days) or T-47D cells (after 18 days) with combinatorial treatments with 1μg ml^{-1} DOXY and the indicated drugs.
(R) Whole body images of mice from Figure 6I shown 5 weeks after xenograft inoculation (n=5).
(S) Working model of the glucose-dependent regulation of VNGD and resulting effects on DNA repair.
(A–R) Results are representative of three independent experiments except as individually marked. Data are mean ± SD, n = 3, *p < 0.05; **p < 0.01; ***p < 0.001; ns, not significant, two-tailed paired Student’s t test (except Fig S6A as described).

Table S1. Selectd lncRNA 1-10

Name	Gene Symbol	RNA length	Site
lnc1	XLOC_003400	664	chr3:196636529-196639627
lnc2	RP11-539L10.3	406	chr4:6670728-6673823
lnc3	NR_038262	2303	chr11:565657-568457
lnc4	NR_036501	4639	chr7:32758286-32762924
lnc5	RP11-452I5.2	565	chr17:74747319-74748912
lnc6	RP11-573N10.1	1051	chr13:98233650-98234700
lnc7	RP11-68I3.11	1052	chr17:29644796-29645847
lnc8	RP11-196G18.23	1234	chr1:149861271-149862504
lnc9	AC068039.4	2102	chr2:171773482-171775844
lnc10	RP11-19J5.2	547	chr15:69820756-69822856

Table S2. Fold change of the mRNA levels of DNA damage response and repair factors

Gene Symbol	log2Fold Change	p-value	Gene Symbol	log2Fold Change	p-value
Base excision repair (BER)			Homologous recombination		
UNG	-0.07559	0.08579	RAD51	0.00770	0.92653
SMUG1	-0.13905	0.06625	RAD51B	-0.12151	0.37821
MBD4	0.17287	0.00167	RAD51D	-0.56653	0.00000
TDG	-0.98684	0.00000	HELQ	-0.17990	0.08055

OGG1	-0.01673	0.81482	SWI5	0.05492	0.51326
MUTYH	-0.14038	0.43853	SWSAP1	0.01353	0.96665
NTHL1	0.18704	0.14879	ZSWIM7	0.15972	0.20703
MPG	0.24892	0.00143	SPIDR	0.05805	0.26887
NEIL1	-0.12956	0.49730	PDS5B	0.11972	0.00482
NEIL2	0.27656	0.00009	DMC1	-0.04993	0.92982
NEIL3	-0.36078	0.00004	XRCC2	0.09791	0.32983
**Other BER and strand break joining factors**			XRCC3	0.44307	0.00000
APEX1	-0.23297	0.00000	RAD52	-0.00040	0.99709
APEX2	-0.30522	0.00000	RAD54L	0.22821	0.00016
LIG3	0.04169	0.41767	RAD54B	-0.17065	0.21194
XRCC1	-0.16771	0.00211	BRCA1	-0.45072	0.00000
PNKP	-0.10226	0.08241	BARD1	-0.06104	0.24297
APLF	0.47822	0.01668	ABRAXAS1	0.15759	0.09417
HMCES	0.27863	0.00000	PAXIP1	-0.16557	0.46403
**Poly(ADP-ribose) polymerase (PARP) enzymes that bind to DNA**			SMC5	-0.14260	0.00013
PARP1	-0.25982	0.00000	SMC6	-0.00220	0.96539
PARP2	-0.16417	0.00026	SHLD1	-0.29132	0.12828
PARP3	0.18470	0.02279	SHLD2	0.51749	0.00000
PARG	0.04557	0.43001	SHLD3	-0.17218	0.26836

PARPBP	-0.02062	0.74582	SEM1	0.04345	0.34741
**Direct reversal of damage**			RAD50	-0.07893	0.58397
MGMT	-0.18925	0.34316	MRE11A	-0.15308	0.00523
ALKBH2	-0.11137	0.24734	NBN	0.30763	0.00000
ALKBH3	-0.15616	0.07205	RBBP8	-0.11815	0.00751
**Repair of DNA-protein crosslinks**			MUS81	-0.01333	0.85766
TDP1	0.46160	0.00000	EME1	0.00998	0.92179
TDP2	0.28968	0.00000	EME2	-0.30408	0.01630
SPRTN	-0.08757	0.27246	SLX1A	1.84412	0.27500
**Mismatch excision repair (MMR)**			SLX1B	-1.01196	0.13848
MSH2	0.02842	0.41295	GEN1	-0.42923	0.00000
MSH3	0.15584	0.03138	**Fanconi anemia**		
MSH6	-0.34789	0.00000	FANCA	-0.18688	0.00122
MLH1	0.21090	0.00000	FANCB	-0.15236	0.21136
PMS2	-0.06481	0.29225	FANCC	0.11670	0.04284
MSH4	0.68743	0.27968	BRCA2	-0.02625	0.62639
MSH5	-0.29460	0.11826	FANCD2	-0.31057	0.00000
MLH3	0.21675	0.00408	FANCE	-0.30844	0.00038
PMS1	0.11213	0.14081	FANCF	-0.45853	0.00000
PMS2P3	0.82579	0.00000	FANCG	0.02497	0.64049
HFM1	-0.15321	0.67351	FANCI	0.18525	0.00000
**Nucleotide excision repair (NER)**			BRIP1	-0.33490	0.00000

XPC	0.38047	0.00000	FANCL	0.09125	0.15574
RAD23B	0.19258	0.00000	FANCM	0.01596	0.80020
CETN2	-0.15979	0.00317	PALB2	0.15842	0.03708
RAD23A	-0.15309	0.00215	RAD51C	0.64220	0.00000
XPA	-0.40048	0.00057	SLX4	-0.42678	0.00000
DDB1	-0.12861	0.00001	FAAP20	0.20005	0.00062
DDB2	0.18066	0.00001	FAAP24	-0.15809	0.19482
RPA1	0.25794	0.00000	FAAP100	0.18098	0.00058
RPA2	0.39830	0.00000	UBE2T	-0.11576	0.07672
RPA3	-0.13520	0.00402	**Non-homologous end-joining**		
ERCC3	0.18331	0.00004	XRCC6	0.14297	0.00002
ERCC2	0.10750	0.04850	XRCC5	-0.06033	0.08698
GTF2H1	0.49227	0.00000	PRKDC	0.22974	0.00000
GTF2H2	0.01400	0.89443	LIG4	-0.28617	0.00189
GTF2H3	0.22539	0.00002	XRCC4	0.60390	0.00000
GTF2H4	-0.09255	0.52579	DCLRE1C	0.00785	0.90608
GTF2H5	0.00930	0.89453	NHEJ1	-0.22473	0.08013
GTF2E2	-0.01488	0.80397	**Modulation of nucleotide pools**		
CDK7	0.30211	0.00000	NUDT1	0.30750	0.00000
CCNH	0.25071	0.00039	DUT	-0.17958	0.00001
NMNAT1	0.84453	0.00000	RRM2B	0.83863	0.00000
ERCC5	-0.43356	0.05707	PARK7	0.11670	0.00086

ERCC1	0.39774	0.00000	DNPH1	0.05811	0.57339
ERCC4	-0.98281	0.00000	NUDT15	0.35335	0.00000
LIG1	-0.03156	0.42655	NUDT18	-0.53615	0.11267
ERCC8	-0.14924	0.06603	**Ubiquitination and modification**		
ERCC6	0.00851	0.89616	UBE2A	-0.05741	0.30287
UVSSA	-0.46697	0.00002	UBE2B	0.20140	0.00160
XAB2	-0.16410	0.00017	RAD18	-0.30819	0.00000
MMS19	0.14988	0.00586	SHPRH	-0.10244	0.16661
**DNA polymerases**			HLTF	-0.10172	0.03968
POLA1	-0.30095	0.00000	RNF168	-0.07292	0.22887
POLB	0.18166	0.04295	RNF8	0.02822	0.69407
POLD1	0.12601	0.00060	RNF4	-0.41319	0.00000
POLD2	0.12959	0.00116	UBE2V2	0.37240	0.00000
POLD3	-0.31443	0.00000	UBE2N	0.22611	0.00000
POLD4	-0.19030	0.14665	USP1	-0.24546	0.00000
POLE	-0.26802	0.00048	WDR48	-0.84788	0.00000
POLE2	-0.41496	0.00000	HERC2	0.18331	0.00004
POLE3	0.01909	0.58928	**Genes defective in diseases associated with sensitivity to DNA damaging agents**		
POLE4	0.28975	0.00267	BLM	-0.02804	0.60718
REV3L	0.08696	0.10803	RMI1	-0.00468	0.94716

MAD2L2	-0.08950	0.05330	TOP3A	0.11108	0.04886
REV1	0.23254	0.00000	WRN	-0.19776	0.00021
POLG	0.16958	0.00038	RECQL4	0.12165	0.00295
POLH	0.36489	0.00000	ATM	0.10140	0.32414
POLI	-0.74653	0.00000	MPLKIP	-0.07694	0.24264
POLQ	-0.28471	0.00000	**Other identified genes with known or suspected DNA repair function**		
POLK	0.07871	0.31298	RPA4	0.71014	0.39580
POLL	0.00187	0.98418	PRPF19	0.05150	0.15143
POLM	-0.10562	0.37293	RECQL	0.00312	0.93187
POLN	-0.23645	0.56697	RECQL5	-0.15175	0.05858
PRIMPOL	-0.12795	0.13355	RDM1	0.22509	0.43271
DNTT	-1.46866	0.58840	NABP2	-0.44427	0.00000
**Editing and processing nucleases**			**Other conserved DNA damage response genes**		
FEN1	0.28035	0.00000	ATR	0.03321	0.56603
FAN1	0.27191	0.00012	ATRIP	-0.10894	0.22471
TREX1	NA	NA	MDC1	-0.90270	0.00000
TREX2	-0.02173	0.95735	PCNA	-0.75693	0.00000
EXO1	-0.50913	0.00000	RAD1	0.00680	0.88304
APTX	0.17945	0.00186	RAD9A	-0.04415	0.64375
SPO11	1.86098	0.64508	HUS1	0.23458	0.00324

ENDOV	-0.43585	0.00001	RAD17	-0.05931	0.21860
DNA2	-0.57730	0.00000	CHEK1	-0.18347	0.00084
DCLRE1A	-0.06847	0.32192	CHEK2	-0.21847	0.00526
DCLRE1B	-0.54831	0.00000	TP53	0.35254	0.00000
EXO5	-0.09864	0.15244	TP53BP1	-0.08356	0.02744
**Chromatin Structure and Modification**			RIF1	0.10187	0.00373
H2AX	0.21628	0.00000	TOPBP1	0.00066	0.98365
CHAF1A	-0.01198	0.80209	CLK2	-0.13209	0.02594
SETMAR	-0.13160	0.08811	PER1	-0.18304	0.01118
ATRX	0.25439	0.00000			

Table S3. Reagents and antibodies

Reagent	Company	Reagent	Company
STF-31	Selleck	Doxycycline	Takara
2-DG	Selleck	Leptomycin B	MedChemExpress
6-AN	MedChemExpress	Niraparib	MedChemExpress
Rotenone	MedChemExpress	Rucaparib	MedChemExpress
ATP	Thermo Fisher	M2 beads	Sigma-Aldrich
puromycin	TOPSCIENCE	A/G beads	Thermo Fisher

Doxrubicin	MedChemExpress	Streptavidin beads	Invitrogen
MMS	Sigma	Trypsin	Gibco
CPT	Selleck	DMEM	Gibco
CDDP	TOPSCIENCE	FBS	Biological Industries

**Antibody**	**Company**	**Catalog #**	**Species**	**Application**
β-actin	CMCTAG	AT0001	Mouse	Western Blotting
GAPDH	CMCTAG	AT0002	Mouse	Western Blotting
Flag-Tag	Sigma	F-3165	Mouse	Western Blotting
His-Tag	CST	2366	Mouse	Western Blotting
γH2A.X	CST	9718	Rabbit	Western Blotting, Immunostaining
H2A.X	Proteintech	10856-1-AP	Rabbit	Western Blotting
H3K9ac	CST	9649	Rabbit	Western Blotting
H3K14ac	CST	7627	Rabbit	Western Blotting
H3K18ac	CST	13998	Rabbit	Western Blotting
H3K27ac	CST	8173	Rabbit	Western Blotting
H3K56ac	PTM bio	PTM-162	Mouse	Western Blotting
Ac-H3	Millpore	382158	Rabbit	Western Blotting
histone H3	CST	4499	Rabbit	Western Blotting
H4K5ac	CST	8647	Rabbit	Western Blotting

H4K8ac	CST	2594	Rabbit	Western Blotting
H4K12ac	CST	13944	Rabbit	Western Blotting
H4K16ac	CST	13534	Rabbit	Western Blotting
Ac-H4	Millpore	04-557	Rabbit	Western Blotting
histone H4	CST	13919	Rabbit	Western Blotting
HMGB1	Abcam	ab228624	Rabbit	Western Blotting, IP
HDAC1	CST	34589	Rabbit	Western Blotting, IP
HDAC2	CST	57156	Rabbit	Western Blotting
HDAC3	CST	85057	Rabbit	Western Blotting
HDAC4	CST	15164	Rabbit	Western Blotting
HDAC5	CST	98329	Mouse	Western Blotting
HDAC6	CST	7558	Rabbit	Western Blotting
HDAC7	Proteintech	26207-1-AP	Rabbit	Western Blotting
HDAC8	Proteintech	17548-1-AP	Rabbit	Western Blotting
HDAC9	Proteintech	67364-1-Ig	Mouse	Western Blotting
HDAC10	Proteintech	24913-1-AP	Rabbit	Western Blotting
PARP	Santa Cruz	sc-8007	Mouse	Western Blotting
SP1	Proteintech	21962-1-AP	Rabbit	Western Blotting，CHIP
Rad51	Santa Cruz	sc-398587	Mouse	Immunostaining
XRCC4	Proteintech	66621-1-Ig	Mouse	Immunostaining

Table S4. Plasmids

Application	Plasmids			
Lentiviral production	pLKO.1	pRSV-Rev	pCMV-VSV-G	pCMV-Gag-Pol
pLKO.1 constructs	pLKO.1-sh-VNGD-1	pLKO.1-sh-VNGD-2	pLKO.1-sh-lnc2	pLKO.1-sh-lnc6
	pLKO.1-sh-H4C15-1	pLKO.1-sh-H4C15-2	pLKO.1-sh-YY1	pLKO.1-sh-IRF1
	pLKO.1-sh-XBP1	pLKO.1-sh-SP1-1	pLKO.1-sh-SP1-2	pLKO.1-sh-HMGB1
	pLKO.1-sh-HDAC1			
Tet-pLKO.1 constructs	Tet-pLKO.1	Tet-pLKO.1-shVNGD-1	Tet-pLKO.1-shVNGD-2	
pCDH constructs	pCDH-CMV	pCDH-flag-HMGB1	pCDH-flag-HMGB1-A	pCDH-flag-HMGB1-B
	pCDH-flag-HMGB1-A+B	pCDH-flag-HMGB1-B+T	pCDH-VNGD	pCDH-flag-SP1
pGL3 reporters	pGL3-Basic	pGL3-BS1	pGL3-BS1M	pGL3-BS2
	pGL3-BS2M			
Mammalian two-hybrid assays	pBIND-Basic	pBIND-HMGB1	pACT-Basic	pACT-HDAC1

pET-28a-6xHIS	pET-28a-6XHIS-HDAC1			
Other constructs	pMD2.G	psPAX2		

Table S5. shRNA sequences

Name	Application	Sequence (5,-3,)
sh-lnc2-F	plasmid construction	ccggGGTTCTTGGTCTCACTGACTTggatccAAGTCAGTGAGACCAAGAACCtttttg
sh-lnc2-R	plasmid construction	aattcaaaaaGGTTCTTGGTCTCACTGACTTggatccAAGTCAGTGAGACCAAGAACC
sh-lnc6-F	plasmid construction	ccggGTTGGCTGATCTCAAACATGAggatccTCATGTTTGAGATCAGCCAACtttttg
sh-lnc6-R	plasmid construction	aattcaaaaaGTTGGCTGATCTCAAACATGAggatccTCATGTTTGAGATCAGCCAAC
sh-VNGD-1F	plasmid construction	ccggGGATTCAGAGCCGTGCTTATAggatccTATAAGCACGGCTCTGAATCCtttttg
sh-VNGD-1R	plasmid construction	aattcaaaaaGGATTCAGAGCCGTGCTTATAggatccTATAAGCACGGCTCTGAATCC
sh-VNGD-2F	plasmid construction	ccggGAGGACTGTCTGCTCCATACTggatccAGTATGGAGCAGACAGTCCTCtttttg
sh-VNGD-2R	plasmid construction	aattcaaaaaGAGGACTGTCTGCTCCATACTggatccAGTATGGAGCAGACAGTCCTC

sh-H4C15-1F	plasmid construction	ccggGGCGACAATCGGATCTGAAGTggatccACTTCAGATCCGATTGTCGCCtttttg
sh-H4C15-1R	plasmid construction	aattcaaaaaGGCGACAATCGGATCTGAAGTggatccACTTCAGATCCGATTGTCGCC
sh-H4C15-2F	plasmid construction	ccggGAAAGGTTGAATGCTCTAGGTggatccACCTAGAGCATTCAACCTTTCtttttg
sh-H4C15-2R	plasmid construction	aattcaaaaaGAAAGGTTGAATGCTCTAGGTggatccACCTAGAGCATTCAACCTTTC
sh-SP1-1F	plasmid construction	ccggGGAAGTGGAGGCAACATCATTggatccAATGATGTTGCCTCCACTTCCtttttg
sh-SP1-1R	plasmid construction	aattcaaaaaGGAAGTGGAGGCAACATCATTggatccAATGATGTTGCCTCCACTTCC
sh-SP1-2F	plasmid construction	ccggGGATCATCAGGGACCAACTCTggatccAGAGTTGGTCCCTGATGATCCtttttg
sh-SP1-2R	plasmid construction	aattcaaaaaGGATCATCAGGGACCAACTCTggatccAGAGTTGGTCCCTGATGATCC
sh-HMGB1-1F	plasmid construction	ccggAGAAGATGAAGATGAAGAACggatccGTTCTTCATCTTCATCTTCTtttttg
sh-HMGB1-1R	plasmid construction	aattcaaaaaAGAAGATGAAGATGAAGAACggatccGTTCTTCATCTTCATCTTCT
sh-HMGB1-2F	plasmid construction	ccggCCGTTATGAAAGAGAAATGAAggatccTTCATTTCTCTTTCATAACGGtttttg

sh-HMGB1-2R	plasmid construction	aattcaaaaaCCGTTATGAAAGAGAAATGAAggatccTTCATTTCTCTTTCATAACGG
sh-HDAC1-F	plasmid construction	ccggTTCTTAACTTTGAACCATACggatccGTATGGTTCAAAGTTAAGAAtttttg
sh-HDAC1-R	plasmid construction	aattcaaaaaTTCTTAACTTTGAACCATACggatccGTATGGTTCAAAGTTAAGAA

Table S6. PCR primers

Name	Application	Sequence (5,-3,)
qRT-lnc1-F/R	qPCR	ATCCACTCAGCCGGTCCTTG / AACCCATCCGCGCAGTCTAA
qRT-lnc2-F/R	qPCR	CCATGTCCCAAGCACTGCTCTA / GCTGAAGGTCCTGAGGCTGT
qRT-lnc3-F/R	qPCR	CCAAGGACCTCCCAGTGACA / TGGTGTCAGCAAGCCAGAAC
qRT-lnc4-F/R	qPCR	CTCCAGGGCGGGAAGATTGT / CTGGACCTCGCCTGACTGAG
qRT-lnc5-F/R	qPCR	GAGAGAGGACCACAGACCAAT / GGGAGCTAGAAGGCTTTACA
qRT-lnc6-F/R	qPCR	TTGCCACCAAGTCAGTACGC / AGGTGAGGTGAGAGGAAGCC
qRT-lnc7-F/R	qPCR	GGCTGGGAGGATACTGAAGC / ACACTGCCCAACATCTGTCA
qRT-lnc9-F/R	qPCR	CCAACCCGGTGCTCTCTGAA / AGCACATCTTGCACCACCCT
qRT-lnc10-F/R	qPCR	CGTGAAGGAAGCTGCTGAGG / GCCTGACGTGGACCAACAAT
qRT-VNGD-F/R	qPCR and RT-PCR	GAAGGCATTGCACTGAGGCT / TGTCGACCTCATCGTCGGAA
Primer-1-F/R	qPCR	TGGACCGAGCCACTGTATTT / GCTTTGACCGACAGTGTGTT
Primer-2-F/R	qPCR	GGGCTTCCGGATTCTGTAGT / ATCCGCTCGTCGGGATTAAA

STC2-F/R	qPCR	CAACACCTGCTCGACTCCTT / GATAGAGGTTACCCAGCGCC
β-actin-F/R	qPCR and RT-PCR	GACCTGACTGACTACCTCATGAAGAT / GTCACACTTCATGATGGAGTTGAAGG
U6-F/R	qPCR	GCTTCGGCAGCACATATACTAAAAT / CGCTTCACGAATTTGCGTGTCAT
H4C15-F/R	qPCR	CCACCTGATCGGCTTAGAGT / CTGGAGACACCACACAACAC
HMGB1-F/R	qPCR	TGTTGCGAAGAAACTGGGAGA / ATATGCAGCAATATCCTTTTCGTAT

Table S7. Probes and primers

Name	Application	Sequence (5,-3,)
BS1-F	CHIP	AATTAGCCCGGCATGGTGGT
BS1-R	CHIP	AGATGGAGTCTCGATCTGTC
BS2-F	CHIP	GGGAAGCGTTCTCAATTGTCC
BS2-R	CHIP	GGAGGAGGAGGGAGAGGAAA
GAPDH-F	CHIP	TACTAGCGGTTTTACGGGCG
GAPDH-R	CHIP	TCGAACAGGAGGAGCAGAGAGCGA
VNGD-1-sense	Biotin probe	ttgttggaccgagccactgtattttagctc
VNGD-2-sense	Biotin probe	gcaagaggactgtctgctccatactgggag
VNGD-3-sense	Biotin probe	GGATTCAGAGCCGTGCTTATA
VNGD-1-antisense	Biotin probe	gagctaaaatacagtggctcggtccaacaa
VNGD-2-antisense	Biotin probe	ctcccagtatggagcagacagtcctcttgc
VNGD-3-antisense	Biotin probe	TATAAGCACGGCTCTGAATCC

VNGD-antisense-F	RNA FISH	TAATACGACTCACTATAGgattcgctgacgtccatg
VNGD-antisense-R	RNA FISH	gctcaggtccctctcactc
VNGD-probe-F	Northern blotting	taatacgactcactataggcagtttgcggtaactactc
VNGD-probe-R	Northern blotting	ctgtcggtcaaagccgtgc
VNGD-FL-F	in-vitro transcription	agTAATACGACTCACTATAGgtcaTTTCTTTTctgttttcctctcc
VNGD-FL-R	in-vitro transcription	agtttttaaaaataatatttttcgggaaaaa
VNGD-P1-F	in-vitro transcription	agTAATACGACTCACTATAGgtcaTTTCTTTTctgttttcctctcc
VNGD-P1-R	in-vitro transcription	aatacagtggctcggtc
VNGD-P2-F	in-vitro transcription	agTAATACGACTCACTATAGttagctcacacaggagaattc
VNGD-P2-R	in-vitro transcription	gggcctccaaaggacaca
VNGD-P3-F	in-vitro transcription	agTAATACGACTCACTATAGcacatggagctagcaaagt
VNGD-P3-R	in-vitro transcription	agtttttaaaaataatatttttcgggaaaaa
VNGD-P2+3-F	in-vitro transcription	agTAATACGACTCACTATAGttagctcacacaggagaattc
VNGD-P2+3-R	in-vitro transcription	agtttttaaaaataatatttttcgggaaaaa
